# Review of German *Spilomicrus* Westwood (Hymenoptera, Diapriidae, Spilomicrini)

**DOI:** 10.3897/BDJ.12.e114515

**Published:** 2024-01-08

**Authors:** Jeremy Joshua Hübner, Vasilisa Chemyreva

**Affiliations:** 1 Zoologische Staatssammlung München, Munich, Germany Zoologische Staatssammlung München Munich Germany; 2 Zoological Institute, Russian Academy of Sciences, St. Petersburg, Russia Zoological Institute, Russian Academy of Sciences St. Petersburg Russia

**Keywords:** checklist, DNA-barcoding, integrative taxonomy, key, new records, new species, new synonymy, parasitoid wasps

## Abstract

**Background:**

This study provides an integrative taxonomy-based review for the genus *Spilomicrus* Westwood in Germany using DNA barcoding and classic morphology.

**New information:**

*Spilomicrussimplex* Tomsik, 1947 is placed in synonymy with *S.antennatus* Jurine, 1807; *Spilomicrusthomsoni* Kieffer, 1911 is removed from synonymy with *S.hemipterus* Marshall, 1868. A lectotype is designated for *Spilomicrusnigripes* Thomson, 1858. Newly recorded for Germany are the following species: *S.thomsoni* Kieffer, 1911, *S.crassiclavis* Marshall, 1868, *S.lusitanicus* Kieffer, 1910 and *S.diversus* Chemyreva, 2021. Three species, *Spilomicrusbrevimalaris* **sp. nov**., *S.flavecorpus* **sp. nov**. and *S.politus* **sp. nov**. are described as new to science. The 23 DNA-barcodes with species identification present a substantial addition over the previous German checklist. This study aims to update the number of nationwide known *Spilomicrus* species from fifteen to twenty. Furthermore, a new key to identify all European *Spilomicrus* species is provided.

## Introduction

This study provides a review of the diaprid genus *Spilomicrus* (Diapriinae, Spilomicrini) in Germany. Diapriidae are parasitoid wasps that are referred to as a “dark taxon” because they are hyper-diverse and it is assumed that a large proportion of the species diversity remains hidden (Hartop et al. 2022). The genus *Spilomicrus* contains more than 100 described species that are worldwide distributed. As is the case for many dark taxa, it is difficult to identify species of *Spilomicrus* because they are miniscule and depict high levels of sexual dimorphism, as well as intraspecific variation. Additionally, many species have distribution areas that can span over several continents and biogeographic areas. *S.formosus* and *S.stigmaticalis*, for example, are found in Europe, Asia and North America (Masner 1991, Notton 1999, Chemyreva 2021). As a consequence, there are many synonyms that refer to the same species although, for example, Masner (1964), Masner and Muesebeck (1968), Notton 2014 and Chemyreva (2021) have made major contributions to rectify some. Although much taxonomic effort has been recently aimed at the description of new species in the Palaearctic (Chemyreva 2015, Chemyreva 2018), it is believed that most representatives of this genus are still unknown to science (Masner 1991). Moreover, it is assumed that a large proportion of the diaprid fauna is found in the tropics, where not much diaprid research has been conducted up to date.

The most recent diversity evaluation that was conducted for Germany was done so over twenty years ago by Blank (2001). Here, fourteen *Spilomicrus* species were recovered, of which two (*S.basalyformis* and *S.nigripes*) have been synonymised since. Additionally, although *S.nigripes* had already been synonymised 21 years earlier by Nixon (1980), this species was still treated as a valid taxon in Blank’s checklist. Two species, *S.bipunctatus* Kieffer, 1911 and *S.nigriclavis* Marshall, 1868 which have been documented (Tomsik 1947) and even originally described (Kieffer 1911) from Germany, are missing from the checklist. Further single records of species in Germany were established by Notton (1999), such as *S.formosus*, which was not included in the aforementioned checklist either. Ultimately, this means that fifteen species are currently acknowledged as being present in Germany.

Overall, the German diaprid fauna is expected to resemble the European species communities which have been recently examined in detail by Chemyreva (2021). To reduce redundancies, we refer to illustrations in Chemyreva’s aforementioned and Notton’s (1999) work when reporting our findings. Species of *Spilomicrus* can be identified using the generic keys by Nixon (1980) and Masner (1991). Generic and species synonymy is documented in Chemyreva (2021).

In order to tackle this megadiverse “dark taxon”, we take advantage of DNA barcoding (Hebert et al. 2003) which is a method that uses the DNA of specimens for species identification. Every animal species on the planet has highly conserved elements in their (mito-)genome that can be used to identify specimens using their DNA only by comparing those sequences with a reference library. The CO1 barcode is a widely used and reliable proxy to distinguish insect species. Every taxon obtains one (or more) species-specific identifiers, the Barcode Index Number (BINs) that are stored online and are publicly available (Ratnasingham and Hebert 2007). One of the many advantages of DNA barcoding is the possibility of associating different specimens, which may have been erroneously described as separate species, based on high levels of sexual dimorphism, to the same species. Overall, DNA barcoding has been proven to be a reliable, fast and cost-efficient method for species identification. Still, it should not be applied exclusively, as the DNA barcode does not always provide the resolution to display the true taxonomic relationships of diverse and complex species (Raupach et al. 2016). Therefore, classic morphology is crucial for the interpretation of a species hypothesis (Schlick-Steiner et al. 2010). In this study, we apply an integrative and complementary approach to review the genus *Spilomicrus*. In this manner, we are increasing the rigour of the taxonomic study because we are combining the advantages that each method provides on its own.

## Materials and methods

Most of the material was collected in Germany in various collecting events, mainly in Bavaria in the framework of GBOL III: Dark Taxa project. Part of the investigated specimens were taken from the Hilpert collection. All specimens are stored at the Bavarian State Collection of Zoology in Munich. In addition, type material from various museums was examined. For species identification, we applied an integrative taxonomy approach, using all resources possible: barcoded and non-barcoded material, as well as genetic and morphological identification methods. Based on the CO1 barcodes which we obtained from the Canadian Centre for DNA Barcoding (CCDB, https://ccdb.ca/resources/), a Maximum-Likelihood tree was calculated using IQ TREE (online tool, Trifinopoulos et al. (2016)) with a subset of 45 *Spilomicrus* sequences and *Labolipsinnupta* as an outgroup to display (Suppl. material 1). The tree was edited using FigTree version 1.4.4 (Rambaut 2010) and Inskape version 1.1.1 (2021, available from: https://inkscape.org/de). All barcoding data (628 records) are stored and accessible in the dataset DS-SPILO ([will be published when accepted]) online on the BOLD platform (www.barcodinglife.org). A table of localities with detailed information on each specimen is attached and also online available on the GBIF platform (https://www.gbif.org/dataset/62c523f3-f065-4677-8124-8cf9b56dd8fb and Suppl. material 2). All the examined type material is listed in the Taxon treatments. We conducted BIN distance analyses (the so-called “Barcode Gap”) to examine how molecularly close questionable MOTUs are with MEGA11 (Tamura et al. 2021) and Assemble Species by Automatic Partitioning (ASAP; Puillandre et al. (2021)). DNA barcodes were obtained from BOLD on the 20 Sept 2023.

The morphological terminology and abbreviations follow Hymenoptera Anatomy ontology (Yoder 2010); the measurements follow Yoder (2004), Chemyreva (2015) and Chemyreva (2018). The general distribution of species was obtained and updated from Notton (1999), Blank (2001) and Chemyreva (2021). The new records are marked with an asterisk (*). Series of images were taken using an Olympus OM-D camera mounted on a Leica M125 C binocular and stacked using Helicon Focus (Version 8).

The following abbreviations for locations in Germany are used: BW = Baden-Wuerttemberg, BY = Bavaria, HE = Hesse, NRW = North Rhine-Westphalia. Museum acronyms: HNHM – Hungarian Natural History Museum, Budapest, Hungary; MNHN – National Museum of Natural History, Paris, France; MZLU – Lund Museum of Zoology, Sweden; NHRS – Swedish Museum of Natural History, Stockholm, Sweden; MMBC – Moravian Museum, Brno, Czech Republic; ZISP – Zoological Institute of the Russian Academy of Sciences, St. Petersburg, Russia.

## Taxon treatments

### Spilomicrus

Westwood, 1832

526F7547-2623-545B-A1AB-F3E4D9300F67

Type species *Spilomicrusstigmaticalis* Westwood, 1832, by original monotypy.

#### Diagnosis

A detailed diagnosis of the genus was given by Masner (1991) and by Masner and García (2002) and, therefore, we only provide a short diagnosis including the most important features.

Medium-sized (1.5–4.5 mm long) melanic wasps. Head subglobose, with mouthparts in lateral view hypognathous; antenna 13-segmented, in females with clava more- or less abrupt, in males antenna thread-like, A4 modified in almost all species. Mesosoma moderately to distinctly wider than high; scutellum with 2 anterior pits and, in most species, with 2 lateral pits and row of smaller posterior pits along posterior margin; forewing with costal vein tubular to nebulous, submarginal vein tubular, marginal vein relatively short, postmarginal and stigmal veins rudimentary or absent; basal vein rarely tubular, in most species nebulous or absent; other veins, at most, nebulous or absent; legs slender to stout, with or without trochanters. Petiole cylindrical in most species; anterior margin of T2 straight, without median cleft or emargination (rarely with 2 lateral folds filled with pilosity); base of S2 arcuate, with moderate to strong cushion of pilosity.

The following part lists all the *Spilomicrus* species found within the framework of the GBOL III project. In comparison to the whole European *Spilomicrus* fauna, three species could not be recorded for Germany and are, therefore, not documented here: *S.sanbornei* Masner, 1991, *S.cursor* Kieffer, 1911 and *S.latus* Chemyreva, 2021. In addition to the morphology, we provide the barcoding information in the form of the BINs and, if necessary, genetic distances for closely-associated taxa. Illustrations are given for the newly-described taxa and the closest sister taxa for a better understanding of the morphological characters and differences. All other species have already been well described and illustrated in Chemyreva (2021).

### Spilomicrus abnormis

Marshall, 1868

56A037BC-9DA5-5CC0-AC54-FB6130ADFB58

AEP5852

Spilomicrus abnormis Marshall, 1868 : 202.
Spilomicrus minimus Kieffer, 1911. Synonymised by Nixon (1980).

#### Description

Illustrated in Chemyreva (2021): fig. 1.

#### Distribution

Czech Republic, Germany*, Hungary, Ireland, Korea, Moldova, Netherlands, Poland, Russia.

### Spilomicrus annulicornis

Kieffer, 1911

A3B1ADFE-8849-52B2-BC27-E0AAF5725F29

ADF4870

Spilomicrus annulicornis Kieffer, 1911 : 788.

#### Description

Illustrated in Chemyreva (2021): fig. 2.

#### Distribution

Austria, Czech Republic, Finland, France, Germany, Netherlands, Russia (European part), United Kingdom.

### Spilomicrus antennatus

(Jurine, 1807)

EAFD5768-17C2-57BF-9C9C-4104E9E0EE2F

AEE0914

**Figure 1.**
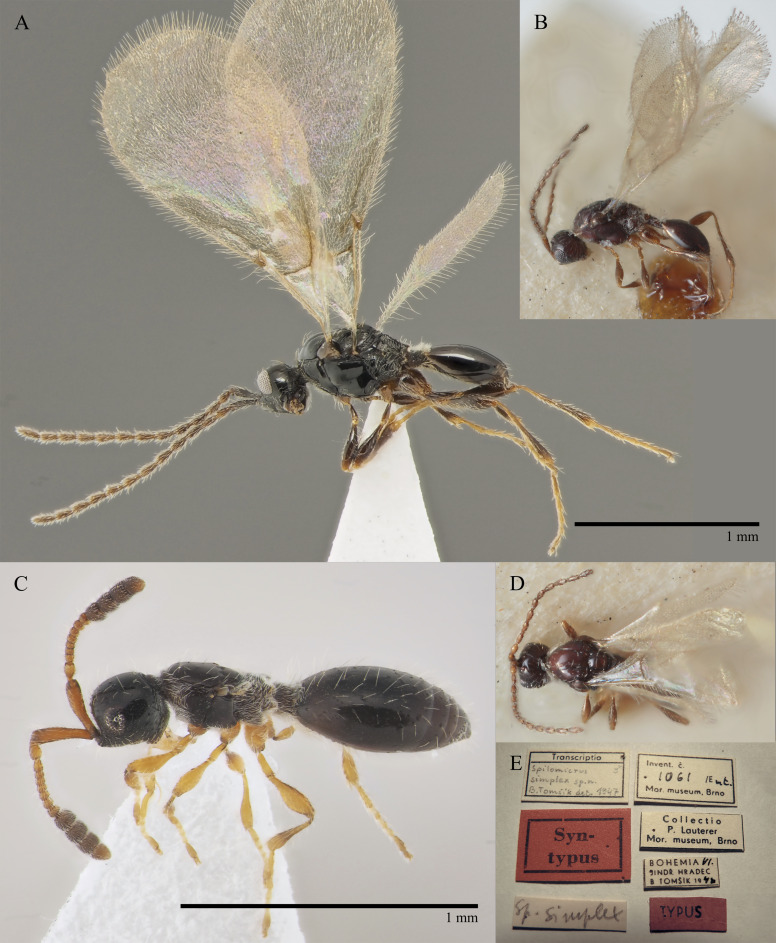
*Spilomicrusantennatus*. **A** male lateral; **B** lectotype *S.simplex* lateral; **C** *S.antennatus* female lateral; **D** lectotype *S.simplex* dorsal **E** corresponding labels.

Psilus antennatus Jurine, 1807 : 319.
Basalys californica Ashmead, 1893. Synonymised by Masner (1991).
Eriopria nigra Kieffer, 1910. Synonymised by Notton (2004).
Eriopria rufithorax Kieffer, 1910. Synonymised by Notton (2004).
Scutellipria quinquepunctata Szabo, 1961. Synonymised by Chemyreva (2021).
Spilomicrus simplex Tomsik, 1947 : 33, 34, 40. **Syn. nov.** Fig. 1B, D and E

#### Materials

- **Type status:** Lectotype. **Occurrence:** catalogNumber: 1061/Ent; recordedBy: P. Laurer; individualCount: 1; sex: male; lifeStage: adult; occurrenceID: 698B71B1-AA08-5BC3-BF96-06C746452B4A; **Taxon:** scientificName: Spilomicussimplex Tomsik, 1947; kingdom: Animalia; phylum: Arthropoda; class: Insecta; order: Hymenoptera; family: Diapriidae; genus: Spilomicrus; specificEpithet: antennatus; scientificNameAuthorship: (Jurine, 1807); **Location:** continent: Europe; **Identification:** identifiedBy: V. Chemyreva I J. Huebner; dateIdentified: 2023; identificationRemarks: designated by Chemyreva (2021), Fig. 1B, D, E; **Event:** eventDate: 1946; **Record Level:** ownerInstitutionCode: MMBC

#### Distribution

Austria, Bulgaria, Czech Republic, Germany, Hungary, Romania, Slovakia, Switzerland, United Kingdom, United States.

#### Notes

What was already suspected by some researchers could be established using DNA barcoding of specimens of each sex (only one female was available, but numerous males). Our obtained sequences were too short to be included in the attached tree (Suppl. material 1), but a female specimen, collected at the Institute's garden in Munich was sequenced and stored in the framework of the Global Malaise trap project (project code GMGMW in BOLD) (Sones et al. 2023). The average genetic distance in between all examined specimens was 0.27%. The common *Spilomicussimplex* Tomsik, that was only described as a male (Fig. 1B, D and E) is a junior synonym of *S.antennatus* (Jurine), which was only described as a female (Fig. 1C).

### Spilomicrus bipunctatus

Kieffer, 1911

D491F7DF-A639-54D5-89E6-63D810782AED

AEC7259

Spilomicrus bipunctatus Kieffer, 1911 : 284, 289.

#### Description

Illustrated in Chemyreva (2021): fig. 4.

#### Distribution

Azerbaijan, Czech Republic, Estonia, France, Germany, Hungary, Italy, Moldova, Netherlands, Poland, Russia (European part), Slovakia, Ukraine, United Kingdom.

### Spilomicrus brevimalaris

Huebner & Chemyreva sp. nov.

5482D19E-9DA2-5F61-B321-8CEE5C6C3753

AEC2138

F47D7379-D468-424F-A72E-97CE9D66C116

#### Materials

- **Type status:** Holotype. **Occurrence:** catalogNumber: ZSM-HYM-33100-G04; recordedBy: Huebner & Chemyreva; individualCount: 1; sex: male; lifeStage: adult; otherCatalogNumbers: BOLD:AEC2138; occurrenceID: CBACBA07-062E-5C74-B9EE-8C7847C5FED0; **Taxon:** scientificName: Spilomicrusbrevimalaris; kingdom: Animalia; phylum: Arthropoda; class: Insecta; order: Hymenoptera; family: Diapriidae; genus: Spilomicrus; specificEpithet: brevimalaris; scientificNameAuthorship: Huebner & Chemyreva, 2023; **Location:** continent: Europe; country: Germany; stateProvince: Bavaria; locality: Ammergau Alps; verbatimElevation: 901; decimalLatitude: 47.606; decimalLongitude: 10.841; **Identification:** identifiedBy: V. Chemyreva I J. Huebner; dateIdentified: 2023; **Event:** eventID: dv.hale1.05; samplingProtocol: malaise trap; eventDate: 18-Jul-2016; **Record Level:** ownerInstitutionCode: SNSB-ZSM
- **Type status:** Paratype. **Occurrence:** catalogNumber: BC-ZSM-HYM-25934-G09; recordedBy: Huebner & Chemyreva; individualCount: 1; sex: female; lifeStage: adult; otherCatalogNumbers: BOLD:AEC2138; occurrenceID: E02F385D-99B0-5BFB-B947-7FE75E5C9CD1; **Taxon:** scientificName: Spilomicrusbrevimalaris; kingdom: Animalia; phylum: Arthropoda; class: Insecta; order: Hymenoptera; family: Diapriidae; genus: Spilomicrus; specificEpithet: brevimalaris; scientificNameAuthorship: Huebner & Chemyreva, 2023; **Location:** continent: Europe; country: Germany; stateProvince: Bavaria; locality: Ammergau Alps; verbatimElevation: 1430; decimalLatitude: 47.5718; decimalLongitude: 10.8807; **Identification:** identifiedBy: V. Chemyreva I J. Huebner; dateIdentified: 2023; **Event:** eventID: dd.amg9.02; samplingProtocol: malaise trap; eventDate: 22-Jul-2015; **Record Level:** ownerInstitutionCode: SNSB-ZSM
- **Type status:** Paratype. **Occurrence:** catalogNumber: ZSM-HYM-33108-G09; recordedBy: Huebner & Chemyreva; individualCount: 1; sex: female; lifeStage: adult; otherCatalogNumbers: BOLD:AEC2138; occurrenceID: 9BF2145E-DC54-5FE1-AF53-FCC9ADB054C3; **Taxon:** scientificName: Spilomicrusbrevimalaris; kingdom: Animalia; phylum: Arthropoda; class: Insecta; order: Hymenoptera; family: Diapriidae; genus: Spilomicrus; specificEpithet: brevimalaris; scientificNameAuthorship: Huebner & Chemyreva, 2023; **Location:** continent: Europe; country: Germany; stateProvince: Baden-Wuerttemberg; locality: Malsch; verbatimElevation: 120; decimalLatitude: 48.884; decimalLongitude: 8.32; **Identification:** identifiedBy: V. Chemyreva I J. Huebner; dateIdentified: 2023; **Event:** eventID: dd.mgart2.13; samplingProtocol: malaise trap; eventDate: 16-Aug-2020; **Record Level:** ownerInstitutionCode: SNSB-ZSM

#### Description

**Male.** Body length 1.4 mm; forewings reaching far beyond apex of metasoma; antenna 0.9 times as long as body.

**Figure 2.**
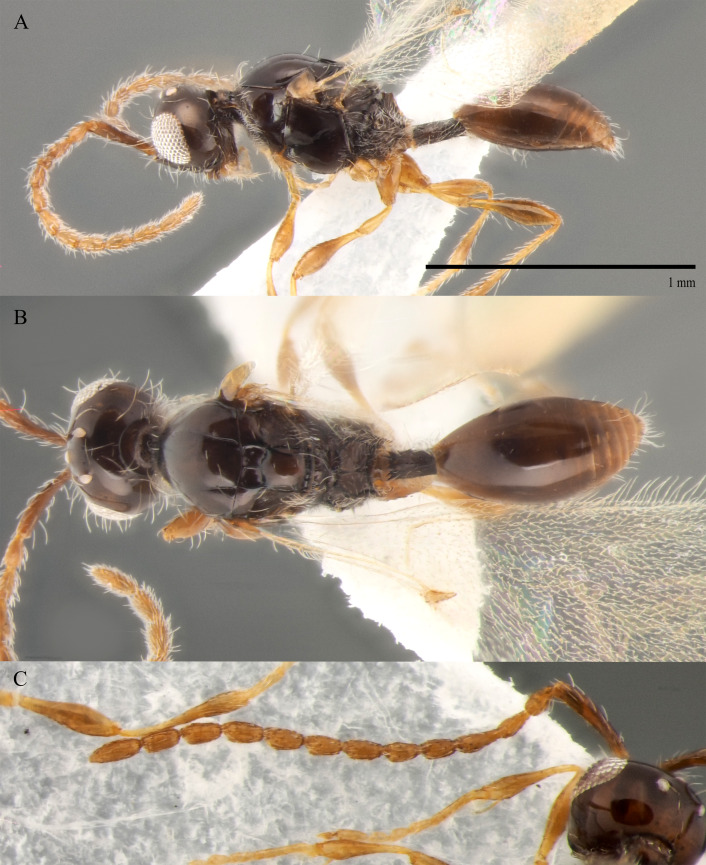
Male holotype *Spilomicrusbrevimalaris* sp. nov. (ZSM-HYM-33100-G04; BOLD:AEC2138). **A** lateral; **B** dorsal; **C** antenna.

**Head**: black; in dorsal view 1.35 times as wide as long, as wide as mesosoma. Temples behind eyes gradually receding posteriorly. Tentorial pit tiny. Malar sulcus absent. Clypeus weakly convex, oval, 1.7 times as wide as high. Mandible reddish-brown, elongate, with upper tooth slightly shorter than lower tooth. Palpi yellow. Eye oval, with scattered long setae; 0.6 times as high as head and 3.8 times as high as malar space. Frons above base of toruli smooth. Postgenal cushion scanty (Fig. 2). **Antennae**: A1 slightly curved, smooth; its apical rim with small lamellae. A2 not compressed. A2–A13 brown, A13 1,3 times as long as A12. Antennomeres length to width ratios in lateral view as in Fig. 2C. **Mesosoma**: dark brown, as wide as high. Neck bare, with shallow longitudinal grooves. Pronotum with median area and pronotal corner pubescent, pronotal cushion dense; pronotal corner weakly prominent, rounded; lateral area of pronotum smooth, bare medially. Tegula brown, large. Mesoscutum convex, 1.2 times as wide as long. Humeral sulcus distinct and narrow. Scutellum slightly convex. Anterior scutellar pits large, circular, smooth inside, with narrow septum. Axillar depression finely pubescent and smooth. Lateral scutellar pit broad. Posterior scutellar pits distinct. Mesopleuron shining bare and smooth, with subalar ridge, longitudinal wrinkles postero-ventrally above middle coxa and sculpture around epicnemial pit. Epicnemial pit tiny, without pubescence inside. Sternaulus absent. Ventral side of mesopleuron scarcely pubescent. Metanotum pubescent, finely sculptured, with three weakly-projecting keels on metascutellum. Propodeum entirely pubescent and coarsely rugose. Median propodeal keel in lateral view projecting into high spine anteriorly (Fig. 2A). All legs slender, pale brown with separated trochantelli. **Wings**: Stigmal vein as long as width of marginal vein. Costa, basal and cubital veins sclerotised and weakly pigmented. **Metasoma**: Petiole 1.9 times as long as wide, cylindrical, entirely longitudinally grooved. Petiole pubescent ventrally and dorsally in anterior part. T2 2.8 times as long as petiole, mainly bare and smooth, with small bunch of setae laterally at anterior margin. T3–T5 sparsely pubescent with semi-erect long setae, smooth. T6 small, setose and bare. T7 tapered, setose. S3–S7 with scattered setae, smooth.

**Figure 3.**
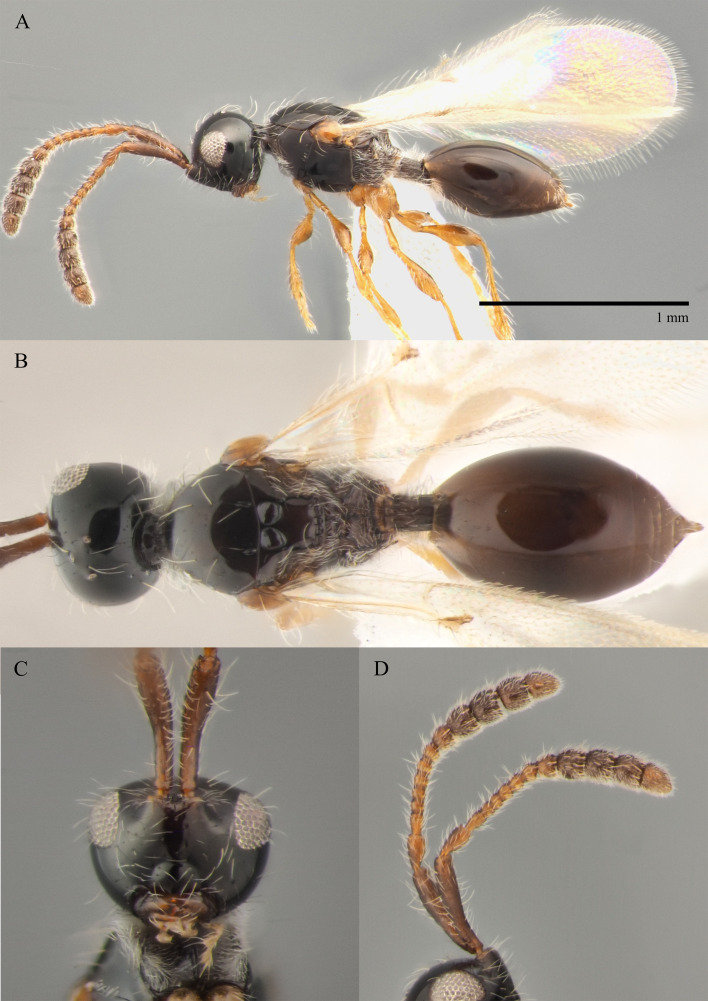
Female paratype *Spilomicrusbrevimalaris* sp. nov. (ZSM-HYM-33108-G09; BOLD:AEC2138). **A** lateral; **B** dorsal; **C** face frontal; **D** antenna.

**Female.** Body length 1.6–1.7 mm. Wings 0.9–1.0 times as long as the body. Pleurostomal distance 0.8 times as long as shortest distance between eyes (Fig. 3C). Malar distance 0.7 times as long as largest diameter of eye. Аntennae brown, clavate with abrupt 5-segmented clava, A13 without ventral pit, A4–A8 moniliform and slightly elongate, A10–A13 with distinct MGS brush ventrally. Scutellum transverse, 0.8 times as long as wide (measured without anterior scutellar pits) (Fig. 3B). Petiole elongate, 1.3–1.4 as long as wide. T2–T8 smooth. S3–S5 smooth. S6 smooth and densely setose. A more detailed description of the female is given by Chemyreva (2021). The females of *S.brevimalaris* sp. nov. were mistakenly described in Chemyreva (2021) as *S.lusitanicus*.

#### Diagnosis

**Figure 4.**
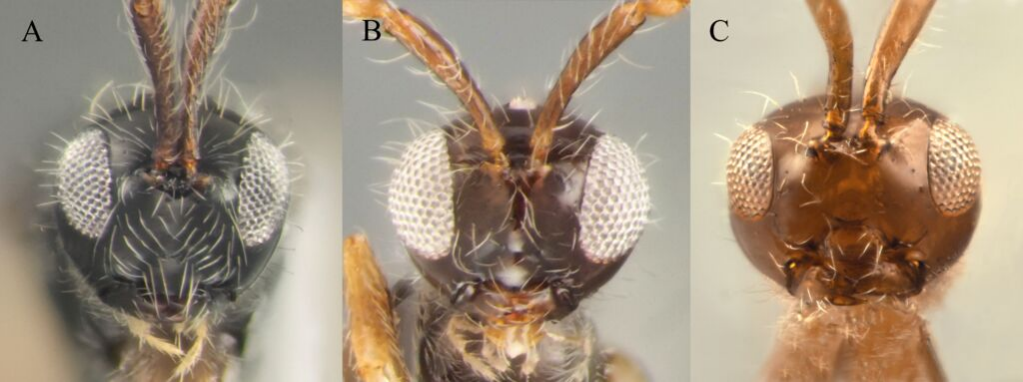
Faces of the males. **A** *Spilomicruslusitanicus*; **B** *Spilomicrusbrevimalaris* **sp. nov.**; **C** *Spilomicrusflavecorpus* **sp. nov.**

**Male.** Body length 1.3–2.1 mm. Face without malar sulcus, pleurostomal distance slightly wider than shortest distance between eyes (Fig. 4B). Malar distance 0.20-0.25 times as long as largest diameter of eye. Front smooth. Аntennae brown, slender and long, with A5–A12 2.0–2.7 times as long as wide in dorsal view. A4 1.1–1.4 times as long as A3 and with keel and emargination reaching 0.55–0.60 of the segment length. Notauli extending to the half of mesoscutum length. Scutellum convex, as long as wide (measured without anterior scutellar pits) (Fig. 2B). Propodeum with weakly-arcuate emargination in dorsal view between plicae. Basal vein and distal part of CU dark and sclerotised. Marginal vein short, less than 1.5 times as long as wide. Petiole elongate, about 1.5–2.0 times as long as wide. T2 pubescent at the base. S8 setose and densely micropunctate.

#### Etymology

The name of this species is a composite Latin masculine adjective derived from “brevis” and “malar” and refers to the short malar distance typical for the males of the new species.

#### Distribution

Germany, Russia (European part). Further BIN records are online available for Italy and Norway. Probably further distributed around western Europe.

#### Notes

The male specimen was used in this case as a holotype, since there is no possibility to use females for the *S.lusitanicus*-species group (both species, *S.brevimalaris* sp. nov. and *S.flavecorpus* sp. nov., are very close to *S.lusitanicus* (Kieffer)). There are two reasons for that: 1) the female for the *S.lusitanicus* is unknown; 2) The most reliable feature to determine this species is the length of the malar space, but this feature does not work for the female determination.

The Russian material that was recorded by Chemyreva (2021) as (the closely related) *S.lusitanicus* actually belongs to *S.brevimalaris* sp. nov.

### Spilomicrus compressus

Thomson, 1859

3388978D-3055-5BCA-B3A5-FC6645C9ED08

ACH2501

Spilomicrus compressus Thomson, 1859 : 369.
Spilomicrus carinatus Kieffer, 1911. Synonymised by Notton (2004).
Spilomicrus crassipes Kieffer, 1911. Synonymised by Notton (2004).

#### Description

Illustrated in Chemyreva (2021): fig. 5.

#### Distribution

Belarus, Czech Republic, Estonia, France, Germany, Hungary, Poland, Russia (European part), Sweden, Ukraine, United Kingdom.

### Spilomicrus crassiclavis

Kieffer, 1911

911508C8-C04B-5A7B-81D1-5CF6748A9163

AEP5849

Spilomicrus crassiclavis Kieffer, 1911 : 788, 797.
Spilomicrus pelion Nixon, 1980. Synonymised by Notton (1999).

#### Description

Illustrated in Notton (1999): figs. 2, 7–9, 17 and 19.

#### Distribution

Czech Republic, Denmark, Finland, Germany*, Japan, Norway, Sweden, United Kingdom.

### Spilomicrus diversus

Chemyreva, 2021

06158B99-4C40-5629-BAA3-030A895BDC56

ADF4749

Spilomicrus diversus Chemyreva, 2021 : 19.

#### Materials

- **Type status:** Holotype. **Occurrence:** recordedBy: Chemyreva; individualCount: 1; sex: female; lifeStage: adult; occurrenceID: 3C510A1D-F303-5A3C-88AA-E130F0615F94; **Taxon:** scientificName: Spilomicrusdiversus; kingdom: Animalia; phylum: Arthropoda; class: Insecta; order: Hymenoptera; family: Diapriidae; genus: Spilomicrus; specificEpithet: diversus; scientificNameAuthorship: Chemyreva, 2021; **Location:** continent: Europe; country: Georgia; stateProvince: Abkhazia; locality: Bzipi River; decimalLatitude: 43.363916; decimalLongitude: 40.495772; **Identification:** identifiedBy: V. Chemyreva; dateIdentified: 2021; **Event:** samplingProtocol: yellow pan trap; eventDate: 11–14-Aug-2015; **Record Level:** ownerInstitutionCode: ZISP
- **Type status:** Paratype. **Occurrence:** recordedBy: Chemyreva; individualCount: 1; sex: female; lifeStage: adult; occurrenceID: BF6D2FE7-2D0B-50DC-9AD9-8E49765AF5DF; **Taxon:** scientificName: Spilomicrusdiversus; kingdom: Animalia; phylum: Arthropoda; class: Insecta; order: Hymenoptera; family: Diapriidae; genus: Spilomicrus; specificEpithet: diversus; scientificNameAuthorship: Chemyreva, 2021; **Location:** continent: Europe; country: Russia; stateProvince: Samara Prov.; locality: Zhigulevskii Nature Reserve; **Identification:** identifiedBy: V. Chemyreva; dateIdentified: 2021; **Event:** eventDate: Jul-28-2009; **Record Level:** ownerInstitutionCode: ZISP
- **Type status:** Paratype. **Occurrence:** recordedBy: Chemyreva; individualCount: 1; sex: male; lifeStage: adult; occurrenceID: D95FF450-21D9-5D16-BD48-C1D871E63B28; **Taxon:** scientificName: Spilomicrusdiversus; kingdom: Animalia; phylum: Arthropoda; class: Insecta; order: Hymenoptera; family: Diapriidae; genus: Spilomicrus; specificEpithet: diversus; scientificNameAuthorship: Chemyreva, 2021; **Location:** continent: Europe; country: Russia; stateProvince: Samara Prov.; locality: Zhigulevskii Nature Reserve; **Identification:** identifiedBy: V. Chemyreva; dateIdentified: 2021; **Event:** eventDate: Jul-28-2009; **Record Level:** ownerInstitutionCode: ZISP
- **Type status:** Paratype. **Occurrence:** recordedBy: K. Tomkovich; individualCount: 1; sex: female; lifeStage: adult; occurrenceID: 41886139-9703-5131-AFE2-DE3224575F4F; **Taxon:** scientificName: Spilomicrusdiversus; kingdom: Animalia; phylum: Arthropoda; class: Insecta; order: Hymenoptera; family: Diapriidae; genus: Spilomicrus; specificEpithet: diversus; scientificNameAuthorship: Chemyreva, 2021; **Location:** continent: Europe; country: Russia; stateProvince: Samara Prov.; locality: Adygea, Belaya River; **Identification:** identifiedBy: V. Chemyreva; dateIdentified: 2021; **Event:** eventDate: 19–24-Aug-2009; **Record Level:** ownerInstitutionCode: ZISP
- **Type status:** Paratype. **Occurrence:** recordedBy: K. Tomkovich; individualCount: 1; sex: female; lifeStage: adult; occurrenceID: 24DE85E8-29C9-5635-8E4B-E2551DC573E8; **Taxon:** scientificName: Spilomicrusdiversus; kingdom: Animalia; phylum: Arthropoda; class: Insecta; order: Hymenoptera; family: Diapriidae; genus: Spilomicrus; specificEpithet: diversus; scientificNameAuthorship: Chemyreva, 2021; **Location:** continent: Europe; country: Russia; stateProvince: Krasnoyarsk Terr.; locality: 70 km of Kryuchkovo Station; **Identification:** identifiedBy: V. Chemyreva; dateIdentified: 2021; **Event:** eventDate: 4–23-Jul-2009; **Record Level:** ownerInstitutionCode: ZISP

#### Diagnosis

**Figure 5.**
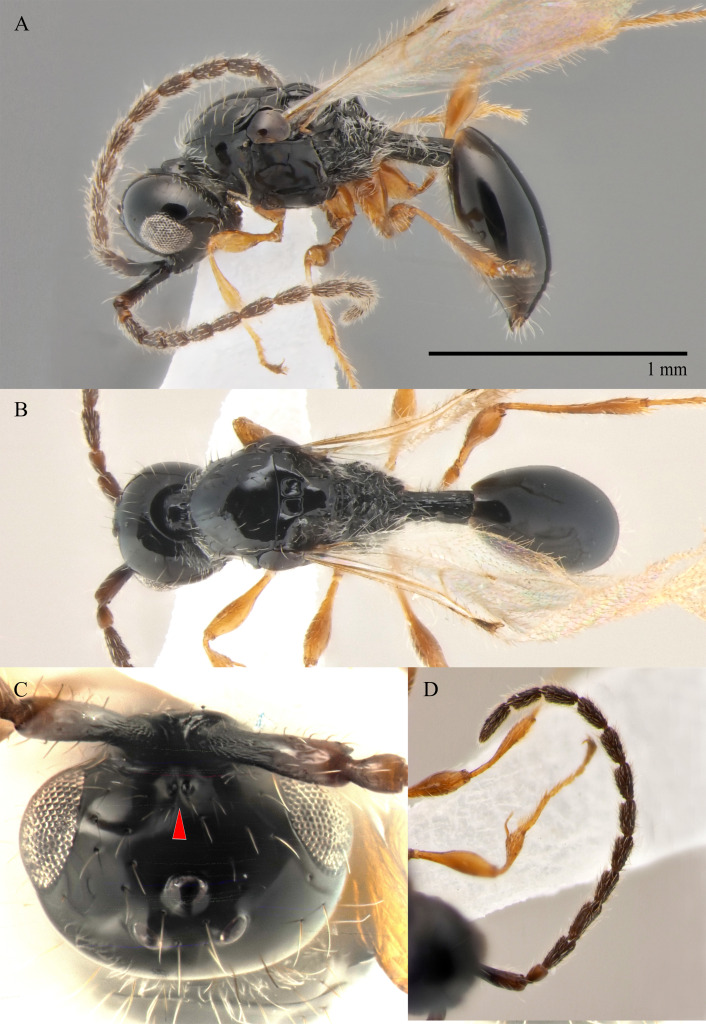
Male *Spilomicrusdiversus* (ZSM-HYM-42367-C03; BOLD:ADF4749). **A** lateral; **B** dorsal; **C** head dorsofrontal, small oval holes marked with red arrow; **D** antenna.

**Figure 6.**
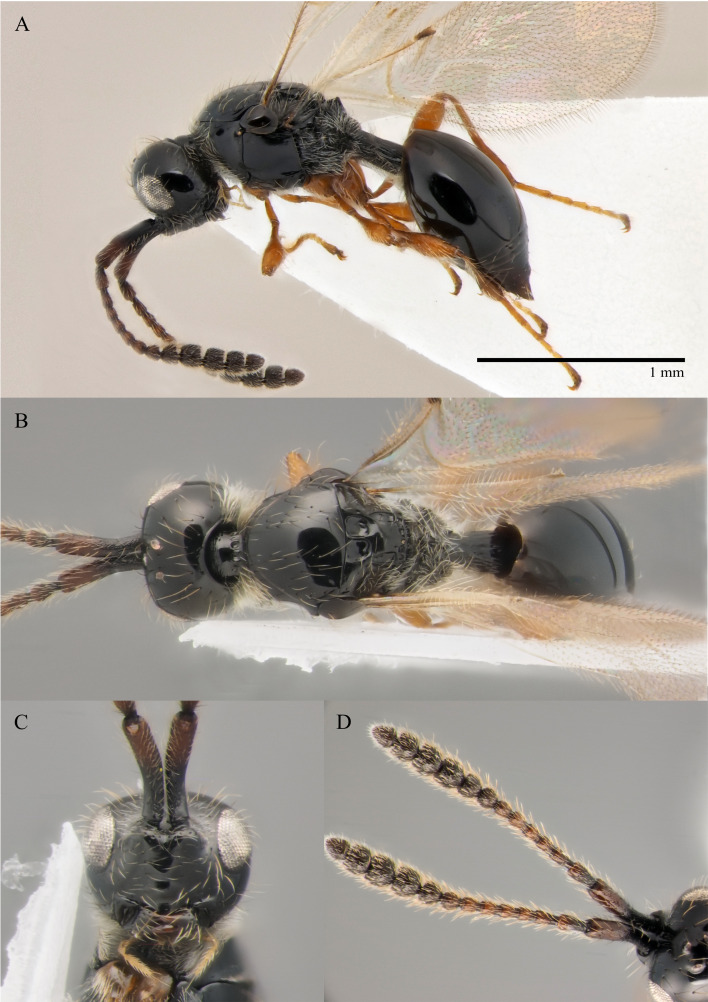
Female *Spilomicrusdiversus* (ZSM-HYM-42318-D01; BOLD:ADF4749). **A** lateral; **B** dorsal; **C** face; **D** antenna dorsal.

**Female.** Face with malar sulcus visible in the form of shallow furrow. Malar distance 0.47 times as long as largest diameter of eye. Front behind scapus with two small holes (as in the male, Fig. 5 C). Head in dorsal view with temples receding behind eyes, as wide as mesosoma. Antennae (Fig. 6 D) with dark abrupt 5-segmented clava, A2–A8 pale brown, A13 narrower than A12, with pit ventrally; A11–A12 about 2.3 times as wide as A5. Notauli absent. Scutellum transverse. Sternaulus smoothed medially and weakly visible anteriorly and posteriorly. Posterior margin of propodeum without arcuate emargination in dorsal view between plicae. Petiole slightly to distinctly elongate. Base of T2 bare.

**Male.** Antennae filiform (Fig. 5D), in dorsal view A5–A12 more than twice as long as wide; A4 slightly longer than A3, with shallow excavation and keel running from base to 0.6 of the segment length. Petiole elongate, at least 1.5 times as long as wide.

#### Distribution

Abkhazia, Czech Republic, Georgia, Germany*, Poland, Russia (European part: Samara Prov., Republic Adygea; Siberia: Krasnoyarskiy Terr.).

#### Notes

Based on new data on intraspecific variability of the *Spilomicrusdiversus* and re-investigation of the type series, we conclude that some specimens should be excluded from the type series. The front sculpture of the specimens from the Far East of Russia (Primorskiy Terr. and Sakhalin Area) is significantly different from both *S.diversus* and *S.politus* sp. nov. and these specimens (paratypes) must be excluded from the type series.

### Spilomicrus flavecorpus

Huebner & Chemyreva sp. nov.

0AFA081D-E26A-5882-AC30-7F9B2BBE5DD5

AAU9373

3E306FCD-F79B-4E1A-968A-B1B1C1E2FF44

#### Materials

- **Type status:** Holotype. **Occurrence:** catalogNumber: ZSM-HYM-33097-H02; recordedBy: Huebner & Chemyreva; individualCount: 1; sex: male; lifeStage: adult; otherCatalogNumbers: BOLD:AAU9373; occurrenceID: D5DD109A-59EF-5A24-8473-55D2E1F44C9E; **Taxon:** scientificName: Spilomicrusflavecorpus; kingdom: Animalia; phylum: Arthropoda; class: Insecta; order: Hymenoptera; family: Diapriidae; genus: Spilomicrus; specificEpithet: flavecorpus; scientificNameAuthorship: Huebner & Chemyreva, 2023; **Location:** continent: Europe; country: Germany; stateProvince: Bavaria; locality: Rhön Mountains; verbatimElevation: 780; decimalLatitude: 50.512; decimalLongitude: 10.069; **Identification:** identifiedBy: V. Chemyreva I J. Huebner; dateIdentified: 2023; **Event:** eventID: dd.kerm1.06; samplingProtocol: malaise trap; eventDate: 26-Jun-2017; **Record Level:** ownerInstitutionCode: SNSB-ZSM
- **Type status:** Paratype. **Occurrence:** catalogNumber: BC-ZSM-HYM-21586-H02; recordedBy: Huebner & Chemyreva; individualCount: 1; sex: female; lifeStage: adult; otherCatalogNumbers: BOLD:AAU9373; occurrenceID: 8B6FA11B-3B86-5434-998A-EFEEAF2E1CCF; **Taxon:** scientificName: Spilomicrusflavecorpus; kingdom: Animalia; phylum: Arthropoda; class: Insecta; order: Hymenoptera; family: Diapriidae; genus: Spilomicrus; specificEpithet: flavecorpus; scientificNameAuthorship: Huebner & Chemyreva, 2023; **Location:** continent: Europe; country: Germany; stateProvince: Bavaria; locality: Bavarian Forest National Park; decimalLatitude: 49.04; decimalLongitude: 13.377; **Identification:** identifiedBy: V. Chemyreva I J. Huebner; dateIdentified: 2023; **Event:** samplingProtocol: malaise trap; eventDate: 15-Jul-2013; **Record Level:** ownerInstitutionCode: SNSB-ZSM
- **Type status:** Paratype. **Occurrence:** catalogNumber: ZSM-HYM-42359-C01; recordedBy: Huebner & Chemyreva; individualCount: 1; sex: female; lifeStage: adult; otherCatalogNumbers: BOLD:AAU9373; occurrenceID: A47590DD-1EC2-5409-AA89-46B91902D85E; **Taxon:** scientificName: Spilomicrusflavecorpus; kingdom: Animalia; phylum: Arthropoda; class: Insecta; order: Hymenoptera; family: Diapriidae; genus: Spilomicrus; specificEpithet: flavecorpus; scientificNameAuthorship: Huebner & Chemyreva, 2023; **Location:** continent: Europe; country: Germany; stateProvince: Bavaria; locality: Marktredwitz; verbatimElevation: 625; decimalLatitude: 50.011; decimalLongitude: 12.044; **Identification:** identifiedBy: V. Chemyreva I J. Huebner; dateIdentified: 2023; **Event:** eventID: 5938_3_For; samplingProtocol: malaise trap; eventDate: 16-Jul-2019; **Record Level:** ownerInstitutionCode: SNSB-ZSM
- **Type status:** Paratype. **Occurrence:** catalogNumber: ZSM-HYM-42363-E04; recordedBy: Huebner & Chemyreva; individualCount: 1; sex: female; lifeStage: adult; otherCatalogNumbers: BOLD:AAU9373; occurrenceID: 150B177D-86F3-56D7-95CE-F9C02E399311; **Taxon:** scientificName: Spilomicrusflavecorpus; kingdom: Animalia; phylum: Arthropoda; class: Insecta; order: Hymenoptera; family: Diapriidae; genus: Spilomicrus; specificEpithet: flavecorpus; scientificNameAuthorship: Huebner & Chemyreva, 2023; **Location:** continent: Europe; country: Germany; stateProvince: Bavaria; locality: Atzmannsberg; verbatimElevation: 550; decimalLatitude: 49.825; decimalLongitude: 11.963; **Identification:** identifiedBy: V. Chemyreva I J. Huebner; dateIdentified: 2023; **Event:** eventID: 6137_4_For; samplingProtocol: malaise trap; eventDate: 11-Jul-2019; **Record Level:** ownerInstitutionCode: SNSB-ZSM

#### Description

**Male.** Body length 1.9 mm; forewings reaching far beyond apex of metasoma; antenna 0.8 times as long as body.

**Figure 7.**
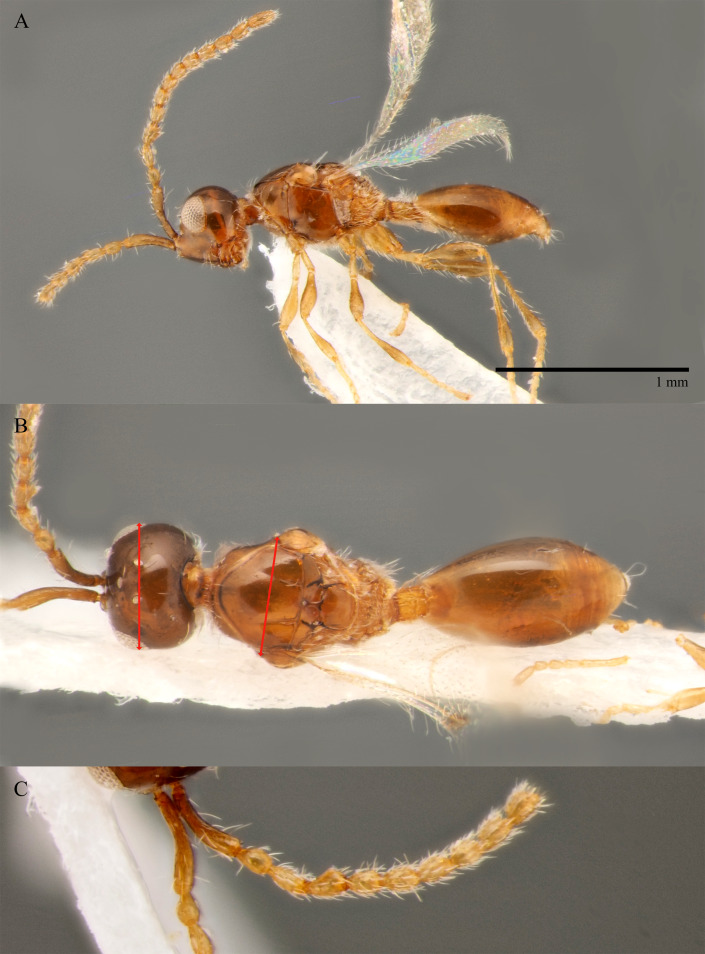
Male holotype *Spilomicrusflavecorpus* sp. nov. (ZSM-HYM-33097-H02; BOLD: AAU9373). **A** lateral; **B** dorsal, head width and mesosoma width marked with arrows; **C** antenna.

**Head**: brown; in dorsal view 1.05 times as wide as long, as wide as mesosoma. Temples behind eyes gradually receding posteriorly. Tentorial pit tiny. Malar sulcus absent. Clypeus weakly convex, oval, 1.85 times as wide as high. Mandible brown, elongate, with upper tooth shorter than lower tooth. Palpi yellow. Eye oval, with few scattered long setae; 0.4 times as high as head and 1.7 times as high as malar space. Frons above base of toruli smooth. Postgenal cushion scanty (Fig. 7A, B). **Antennae**: brown. A1 slightly curved, smooth; its apical rim with small lamellae. A2 not compressed. A13 1.4 times as long as A12. Antennomeres length to width ratios in lateral view as in Fig. 7A, C. **Mesosoma**: brown, 1.1 times as wide as high. Neck with few scattered setae and shallow longitudinal grooves. Pronotum with median area scarcely setose and pronotal corner densely pubescent, pronotal cushion dense; pronotal corner weakly prominent, rounded; lateral area of pronotum smooth, bare medially. Tegula brown, large. Mesoscutum convex, 1.25 times as wide as long. Humeral sulcus distinct and narrow. Scutellum slightly convex. Anterior scutellar pits large, circular, smooth inside, with narrow septum. Axillar depression finely pubescent and smooth. Lateral scutellar pit broad. Posterior scutellar pits small. Mesopleuron shining bare and smooth, with small depression next to epicnemial pit, subalar ridge below tegula and longitudinal wrinkles postero-ventrally above middle coxa. Epicnemial pit tiny, without pubescence inside. Sternaulus absent. Ventral side of mesopleuron scarcely pubescent. Metanotum pubescent, finely sculptured, with three weakly-projecting keels on metascutellum. Propodeum entirely pubescent and finally sculptured. Median propodeal keel in lateral view high raised anteriorly (Fig. 7A). All legs slender, pale brown with separated trochantelli. **Wings**: Stigmal vein as long as width of marginal vein. Costal, basal and cubital veins sclerotised and weakly pigmented. **Metasoma**: Petiole entirely pubescent. T2 4.5 times as long as petiole, mainly bare and smooth, with small bunch of setae laterally at anterior margin. T3–T5 sparsely pubescent with semi-erect long setae, smooth. T6 small, setose and bare. T7 tapered, setose. S3–S7 with scattered setae, smooth.

**Figure 8.**
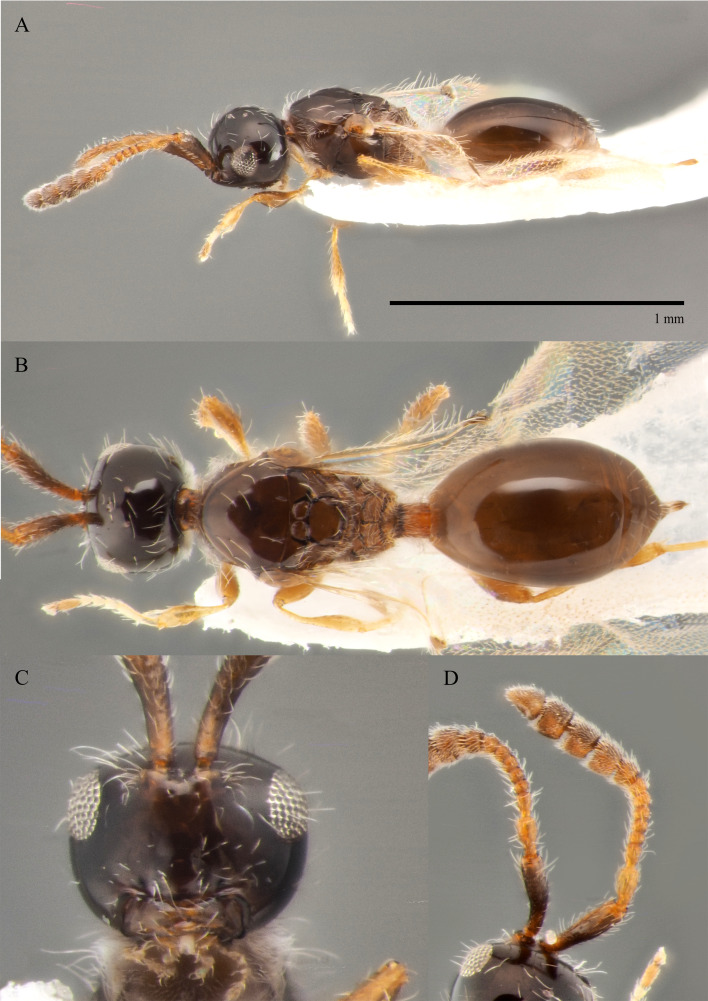
Female paratype *Spilomicrusflavecorpus* sp. nov. (ZSM-HYM-42363-E04; BOLD: AAU9373). **A** lateral; **B** dorsal; **C** face frontal; **D** antenna.

**Female.** Body length 1.7–1.8 mm. Pleurostomal distance 0.74 times as long as shortest distance between eyes (Fig. 8C). Malar distance 0.73 times as long as largest diameter of eye (Fig. 8C). Аntennae brown, clavate with abrupt 5-segmented clava, A13 without ventral pit, A4–A8 moniliform and transverse, A10–A13 with distinct MGS brush ventrally (Fig. 8D). Petiole as long as wide. T2–T6 smooth. T7–T8 weakly punctured. S3–S5 smooth. S6 smooth and densely setose.

#### Diagnosis

**Male.** Face without malar sulcus, pleurostomal distance 0.9 times as wide as shortest distance between eyes (Fig. 4C). Malar distance 0.42 times as long as largest diameter of eye. Front smooth. Аntennae brown, filiform, with A5 1.3 and A12 1.6 times as long as wide in dorsal view. A4 1.1 times as long as A3 and with keel and emargination reaching to half of the segment (Fig. 7C). Notauli marked as short grooves posteriorly (Fig. 7B). Scutellum convex, as long as wide (measured without anterior scutellar pits) (Fig. 7B). Propodeum with weak emargination between plicae. Marginal vein 1.25 times as long as wide. Petiole slightly elongate, about 1.1 times as long as wide. T2 pubescent at the base. S8 setose and densely micropunctate.

#### Etymology

The name of this species is a composite Latin masculine adjective derived from the adverb “flave” (yellowly) and “corpus” and refers to the colouration of the body.

#### Distribution

Germany. Further BIN records are online available for Canada. Probably further distributed around the Palaearctic and Nearctic.

#### Notes

The reason for the selection of the holotype is analogous to that of *S.brevimalaris* sp. nov. (check Notes).

### Spilomicrus flavipes

Thomson, 1858

3A39BFD8-A774-5B26-95AC-A35432F6925E

ACL2543

Spilomicrus flavipes Thomson, 1858: 369.
Spilomicrus szelenyii Szabo, 1977. Synonymised by Chemyreva (2021).

#### Description

Illustrated in Chemyreva (2021): fig. 8.

#### Distribution

Czech Republic, France, Germany, Hungary, Ireland, Moldova, Mongolia, Poland, Russia, Sweden, United Kingdom.

### Spilomicrus formosus

Jansson, 1942

ACFC1E8B-1BE9-550F-83E5-7ECA049A42D7

AAU9811

Spilomicrus formosus Jansson, 1942 : 215.

#### Description

Illustrated in Notton (1999): figs. 3, 4, 10–12, 18 and 20.

#### Distribution

Belgium, Canada, Czech Republic, Denmark, Finland, Germany, Great Britain, Ireland, Japan, Norway, Russia, Slovakia, Sweden, United States.

### Spilomicrus hemipterus

Marshall, 1868

ED472E89-756F-5F97-8188-853291DA3829

ADM6694

**Figure 9.**
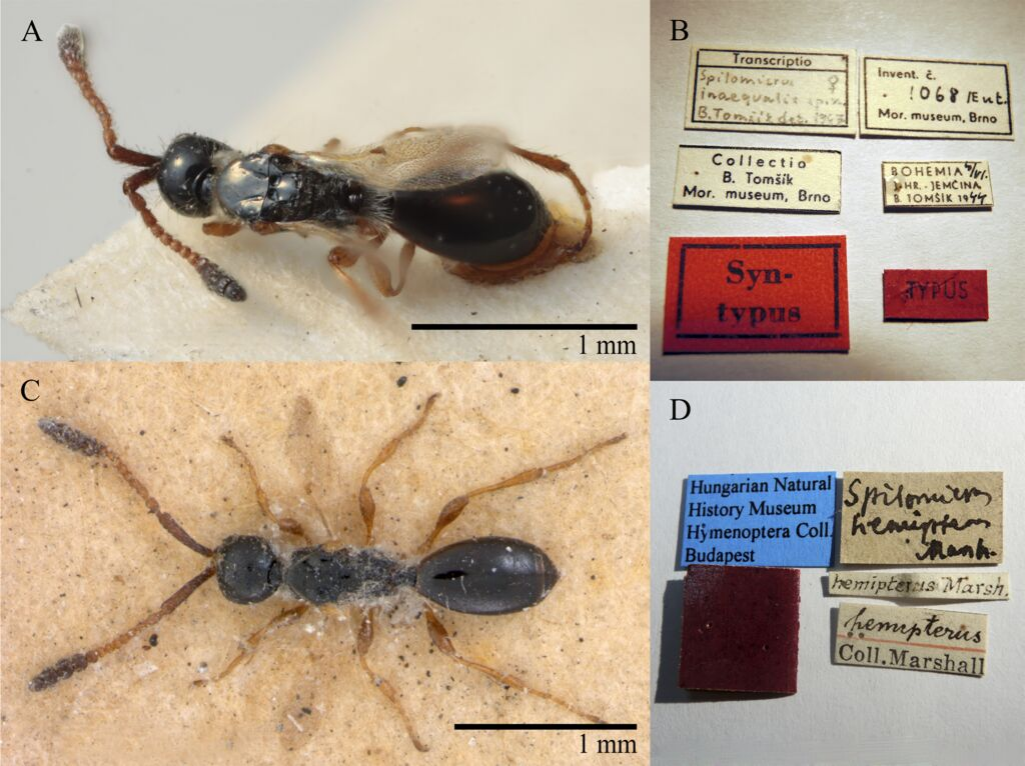
Female lectotypes. **A** *Spilomicrusinaequalis* dorsal; **B** corresponding labels; **C** *Spilomicrushemipterus* dorsal; **D** corresponding labels.

Spilomicrus hemipterus Marshall, 1868 : 202.
Spilomicrus inaequalis Tomsik, 1941: 34, 38, 42. Synonymised by Chemyreva (2021). Fig. 9A and B.
Spilomicrus pedisequus Kieffer, 1916: 784, 787. Synonymised by Nixon (1980).

#### Materials

- **Type status:** Lectotype. **Occurrence:** recordedBy: B. Tomsik; individualCount: 1; sex: female; lifeStage: adult; otherCatalogNumbers: BOLD:ADM6694; occurrenceID: 5CFDD1B7-4855-5101-B094-8EE5657DAC38; **Taxon:** scientificName: Spilomicrushemipterus; kingdom: Animalia; phylum: Arthropoda; class: Insecta; order: Hymenoptera; family: Diapriidae; genus: Spilomicrus; specificEpithet: hemipterus; scientificNameAuthorship: Marshall, 1868; **Location:** continent: Europe; **Identification:** identificationRemarks: designated in Chemyreva (2021), Fig. 17C, D; **Event:** eventDate: 1944; **Record Level:** ownerInstitutionCode: MNHN
- **Type status:** Lectotype. **Occurrence:** recordedBy: Marshall; individualCount: 1; sex: female; lifeStage: adult; otherCatalogNumbers: BOLD:ADM6694; occurrenceID: 3E890838-0263-587C-86F8-418105C938B7; **Taxon:** scientificName: Spilomicrusinaequalis; kingdom: Animalia; phylum: Arthropoda; class: Insecta; order: Hymenoptera; family: Diapriidae; genus: Spilomicrus; specificEpithet: hemipterus; scientificNameAuthorship: Marshall, 1868; **Location:** continent: Europe; **Identification:** identificationRemarks: designated in Chemyreva (2021), Fig. 17A, B; **Record Level:** ownerInstitutionCode: MMBC

#### Diagnosis

Malar sulcus partly developed, shallow; neck of prothorax bare anteriorly; pleurostomal distance distinctly shorter than distance between eyes in front view; temples behind eyes gradually receding posteriorly; male А4 with keel reaching 0.7 of the segment length, A4 0.65 times as long as A3, widened apically; antenna distinctly bicolorous with more abrupt clava; female A9 without or with weakly indicated MGS brush; A13 with distinct small pit ventrally; A13 in dorsal and lateral views narrower than A12; А9 distinctly narrower and shorter than А10; notauli present in the form of broad grooves posteriorly; sternaulus absent; wings reaching to one-fourth of metasoma length to distinctly beyond the apex of metasoma; female petiole elongate, about 1.2 times as long as wide.

#### Distribution

Austria, Croatia, Czech Republic, France, Germany, Hungary, Moldova, Netherlands, Poland, Russia (European part), Switzerland, Ukraine, United Kingdom.

### Spilomicrus integer

Thomson, 1859

956D7D74-D893-586B-802A-515848CA6785

ADF4750

Spilomicrus integer Thomson, 1859 : 369.
Spilomicrus major Vollenhoven, 1879. Synonymised by Chemyreva (2021).

#### Description

Illustrated in Chemyreva (2021): fig. 10.

#### Distribution

Czech Republic, France, Germany, Hungary, Netherlands, Poland, Romania, Russia (European part), Slovakia, Sweden, Ukraine, United Kingdom.

### Spilomicrus lusitanicus

(Kieffer, 1910)

4CC9BA33-490D-57F4-896B-646AC7855F87

AEK2205

**Figure 10.**
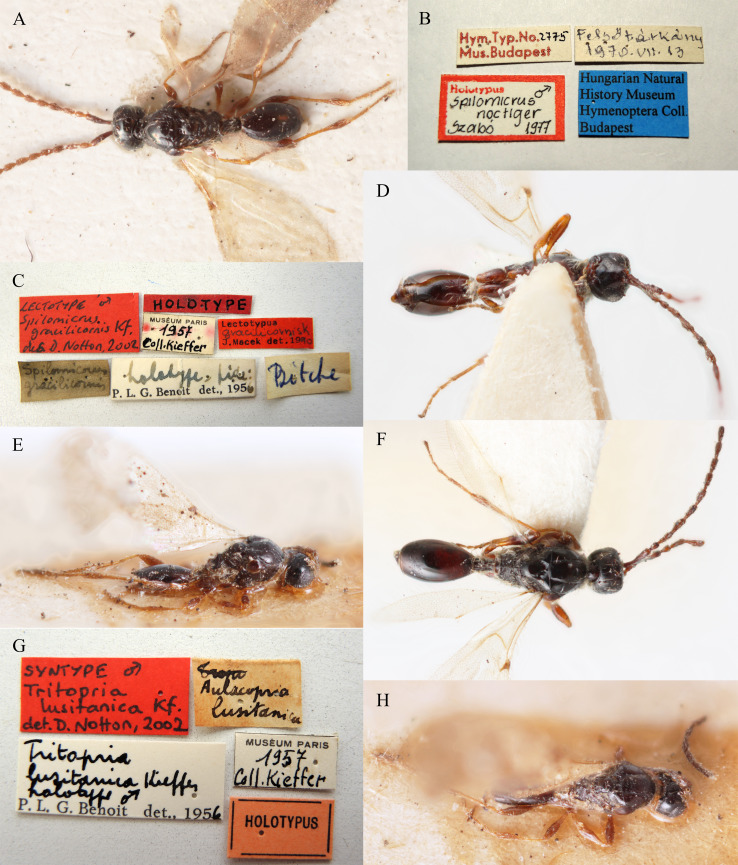
Male types. **A** holotype of *Spilomicrusnoctiger* Szabó; **B** corresponding labels; **D**, **F** lectotype of *Spilomicrusgracilicornis* Kieffer (designated by Notton (2004)); **C** corresponding labels; **E**, **H** lectotype of *Trichoprialusitanica* Kieffer (designated by Chemyreva (2021)); **G** corresponding labels.

Tritopria lusitanica Kieffer, 1910 : 749, male. Fig. 10E, G and H.
Spilomicrus gracilicornis Kieffer, 1911. Synonymised by Chemyreva (2021). Fig. 10C, D and F.
Spilomicrus noctiger Szabo, 1977. Synonymised by Chemyreva (2021). Fig. 10A and B.

#### Materials

- **Type status:** Holotype. **Occurrence:** recordedBy: Kieffer; individualCount: 1; sex: male; lifeStage: adult; occurrenceID: 5274E46C-F7BF-506B-A39B-7027769199D7; **Taxon:** scientificName: Tritopria lusitanicus Kieffer, 1910; kingdom: Animalia; phylum: Arthropoda; class: Insecta; order: Hymenoptera; family: Diapriidae; genus: Spilomicrus; specificEpithet: lusitanicus; scientificNameAuthorship: (Kieffer, 1910); **Location:** continent: Europe; **Identification:** identifiedBy: V. Chemyreva; dateIdentified: 2021; identificationRemarks: designated by Chemyreva (2021), Fig. 11E, G, H; **Event:** eventDate: 1956; **Record Level:** ownerInstitutionCode: MNHN]
- **Type status:** Lectotype. **Occurrence:** recordedBy: Kieffer; individualCount: 1; sex: male; lifeStage: adult; otherCatalogNumbers: BOLD:AEK2205; occurrenceID: 6E75EA16-1397-560F-8A1D-6B47EF00B5D2; **Taxon:** scientificName: Spilomicrusgracilicornis Kieffer, 1911; kingdom: Animalia; phylum: Arthropoda; class: Insecta; order: Hymenoptera; family: Diapriidae; genus: Spilomicrus; specificEpithet: lusitanicus; scientificNameAuthorship: (Kieffer, 1910); **Location:** continent: Europe; **Identification:** identifiedBy: V. Chemyreva; dateIdentified: 2021; identificationRemarks: designated by Chemyreva (2021), Fig. 11C, D, F; **Event:** eventDate: 1956; **Record Level:** ownerInstitutionCode: MNHN
- **Type status:** Holotype. **Occurrence:** catalogNumber: 2775; recordedBy: P. L. G. Benoit; individualCount: 1; sex: male; lifeStage: adult; occurrenceID: 64EB8BCA-C3A7-599F-94FC-B9DBD87BA7EB; **Taxon:** scientificName: Spilomicrusnoctiger Szabo, 1997; kingdom: Animalia; phylum: Arthropoda; class: Insecta; order: Hymenoptera; family: Diapriidae; genus: Spilomicrus; specificEpithet: lusitanicus; scientificNameAuthorship: (Kieffer, 1910); **Location:** continent: Europe; **Identification:** identifiedBy: V. Chemyreva; dateIdentified: 2021; identificationRemarks: designated by Chemyreva (2021), Fig. 11A, B; **Event:** eventDate: Jul-13-1970; **Record Level:** ownerInstitutionCode: HNHM

#### Diagnosis

**Figure 11.**
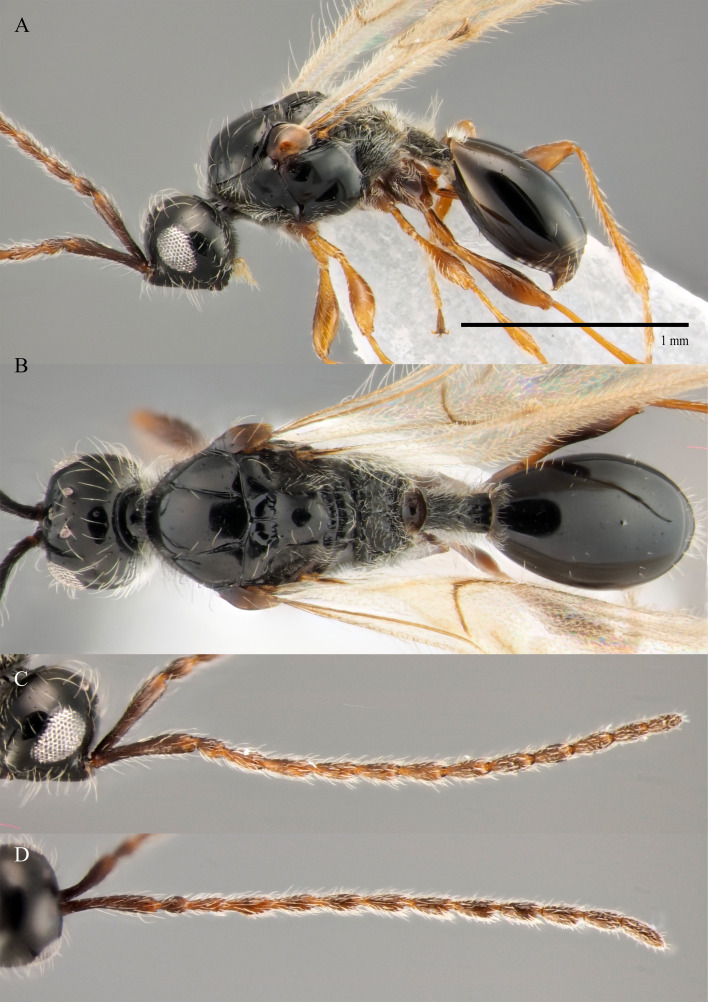
Male *Spilomicruslusitanicus* (ZSM-HYM-42423-H01; BOLD:AEK2205). **A** lateral; **B** dorsal; **C** antenna lateral; **D** antenna dorsal.

**Male.** Body length 1.9–2.5 mm. Face without malar sulcus, pleurostomal distance slightly wider than shortest distance between eyes. Malar distance 0.45–0.55 times as long as largest diameter of eye. Front smooth. Аntennae dark brown, slender and long, with A5–A12 2.3–3.3 times as long as wide. A4 1.2–1.25 times as long as A3 and with keel and emargination reaching to 0.5–0.55 of the segment length (Fig. 11D). Notauli almost complete, but shallow anteriorly (Fig. 11B). Scutellum convex, 1–1.2 times as long as wide (measured without anterior scutellar pits). Propodeum with not deep emargination between plicae in dorsal view. Basal vein and distal part of CU dark and sclerotised. Marginal vein short, less than 1.5 times as long as wide. Petiole elongate, 1.7–1.8 times as long as wide. T2 pubescent at the base. S8 almost smooth, with few setae and very weak elongated wrinkles.

#### Distribution

Algeria, Austria, Czech Republic, France, Germany*, Hungary, Italy*, Portugal, Russia (European part).

#### Notes

The most important features of this species, such as malar and pleurostomal distances cannot be examined in the lectotype of *Tritoprialusitanica* because the face of the type specimen is hidden in glue. However, secondary diagnostic characters (proportions of the remaining antennomeres, width of the head and proportions of the scutellum) lead us to believe that all type specimens belong to a single species and correspond with the examined material mentioned above under the name *Spilomicruslusitanicus*. The females are unknown. The females described by Chemyreva (2021) belong to the *S.brevimalaris* sp. nov.

### Spilomicrus modestus

Tomsik, 1947

29F33E2E-4F49-59D2-B73F-916EE22217EB

AEJ2099

Spilomicrus modestus Tomsik, 1947 : 33, 39, 42.

#### Description

Illustrated in Chemyreva (2021): fig. 13.

#### Distribution

Austria, Czech Republic, Finland, Germany, Hungary, Moldova, Russia (European part and East Siberia), Ukraine.

### Spilomicrus nigriclavis

Marshall, 1868

1B572FE1-63CF-5C03-B367-02C7B133C948

AEK0961

Spilomicrus nigriclavis Marshall, 1868 : 228.
Spilomicrus punctatus Kozlov, 1978 : 591, nom. praeocc., non *Spilomicruspunctatus* (Cameron, 1889).
Spilomicrus kozlovi Notton, 2014. Synonymised by Chemyreva (2021).
Spilomicrus nigriclavis var. armatus Kieffer, 1911 : 781, nom. praeocc., non *Spilomicrusarmatus* (Ashmead, 1893).
Spilomicrus nigriclavis var. subarmatus Kieffer, 1912. Synonymised by Chemyreva (2021).

#### Description

Illustrated in Chemyreva (2021): fig. 14.

#### Distribution

France, Germany, Netherlands, Russia (European part), Sweden, United Kingdom.

### Spilomicrus politus

Huebner & Chemyreva sp. nov.

68138890-B5A4-5FD7-8046-AF366D1A2882

AER1505

ACZ2358

E9C61643-B816-4379-97E5-A71D2603E8B1

#### Materials

- **Type status:** Holotype. **Occurrence:** catalogNumber: ZSM-HYM-42456-C12; recordedBy: Huebner & Chemyreva; individualCount: 1; sex: female; lifeStage: adult; otherCatalogNumbers: BOLD:ACZ2358; occurrenceID: 2C22789C-3D9D-5594-9D9E-71E8AC0ABD87; **Taxon:** scientificName: Spilomicruspolitus; kingdom: Animalia; phylum: Arthropoda; class: Insecta; order: Hymenoptera; family: Diapriidae; genus: Spilomicrus; specificEpithet: politus; scientificNameAuthorship: Huebner & Chemyreva, 2023; **Location:** continent: Europe; country: Germany; stateProvince: Bavaria; locality: Munich; verbatimElevation: 516; decimalLatitude: 48.164; decimalLongitude: 11.497; **Identification:** identifiedBy: V. Chemyreva I J. Huebner; dateIdentified: 2023; **Event:** eventID: gb.botgar1.10; samplingProtocol: malaise trap; eventDate: 01-Sep-2021; **Record Level:** ownerInstitutionCode: SNSB-ZSM
- **Type status:** Paratype. **Occurrence:** catalogNumber: ZSM-HYM-42369-G02; recordedBy: Huebner & Chemyreva; individualCount: 1; sex: female; lifeStage: adult; otherCatalogNumbers: BOLD:ACZ2358; occurrenceID: EF90EABD-F85F-52CF-8EE2-796379EB829F; **Taxon:** scientificName: Spilomicruspolitus; kingdom: Animalia; phylum: Arthropoda; class: Insecta; order: Hymenoptera; family: Diapriidae; genus: Spilomicrus; specificEpithet: politus; scientificNameAuthorship: Huebner & Chemyreva, 2023; **Location:** continent: Europe; country: Germany; stateProvince: Baden-Wuerttemberg; locality: Gaggenau; verbatimElevation: 340; decimalLatitude: 48.821; decimalLongitude: 8.388; **Identification:** identifiedBy: V. Chemyreva I J. Huebner; dateIdentified: 2023; **Event:** eventID: dd.mbach.05; samplingProtocol: malaise trap; eventDate: 21-Aug-2011; **Record Level:** ownerInstitutionCode: SNSB-ZSM
- **Type status:** Paratype. **Occurrence:** catalogNumber: ZSM-HYM-42373-F02; recordedBy: Huebner & Chemyreva; individualCount: 1; sex: female; lifeStage: adult; otherCatalogNumbers: BOLD:ACZ2358; occurrenceID: E1875FF2-D75C-554D-B113-04D0EE157E8C; **Taxon:** scientificName: Spilomicruspolitus; kingdom: Animalia; phylum: Arthropoda; class: Insecta; order: Hymenoptera; family: Diapriidae; genus: Spilomicrus; specificEpithet: politus; scientificNameAuthorship: Huebner & Chemyreva, 2023; **Location:** continent: Europe; country: Germany; stateProvince: Bavaria; locality: Munich; verbatimElevation: 516; decimalLatitude: 48.164; decimalLongitude: 11.497; **Identification:** identifiedBy: V. Chemyreva I J. Huebner; dateIdentified: 2023; **Event:** eventID: gb.botgar1.09; samplingProtocol: malaise trap; eventDate: 11-Aug-2021; **Record Level:** ownerInstitutionCode: SNSB-ZSM
- **Type status:** Paratype. **Occurrence:** catalogNumber: ZSM-HYM-42466-G05; recordedBy: Huebner & Chemyreva; individualCount: 1; sex: female; lifeStage: adult; otherCatalogNumbers: BOLD:ACZ2358; occurrenceID: C7CE6D4E-9617-55DE-A7AD-03ADCFFD1528; **Taxon:** scientificName: Spilomicruspolitus; kingdom: Animalia; phylum: Arthropoda; class: Insecta; order: Hymenoptera; family: Diapriidae; genus: Spilomicrus; specificEpithet: politus; scientificNameAuthorship: Huebner & Chemyreva, 2023; **Location:** continent: Europe; country: Germany; stateProvince: Bavaria; locality: Paehl; verbatimElevation: 720; decimalLatitude: 47.941; decimalLongitude: 11.183; **Identification:** identifiedBy: V. Chemyreva I J. Huebner; dateIdentified: 2023; **Event:** eventID: dd.pmor5.06; samplingProtocol: malaise trap; eventDate: 27-Aug-2020; **Record Level:** ownerInstitutionCode: SNSB-ZSM

#### Description

**Figure 12.**
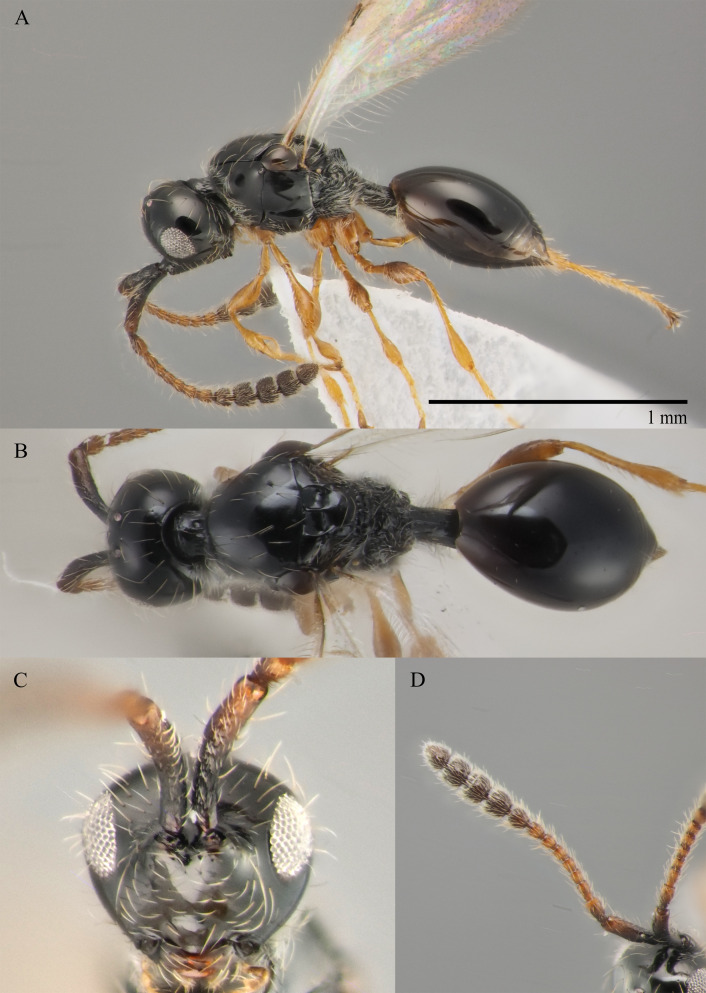
Female holotype *Spilomicruspolitus* sp. nov. (ZSM-HYM-42456-C12; BOLD: ACZ2358). **A** lateral; **B** dorsal; **C** face; **D** antenna dorsal.

**Female (holotype)**. Body length 1.8 mm; forewing extending far beyond apex of metasoma; antenna 0.68 times as long as body. **Head**: black, in dorsal 0.95 times as wide as metasoma. Tentorial pits absent. Clypeus weakly convex, 0.6 times as high as wide. Mandible dark brown, elongate, its upper tooth slightly shorter than lower tooth. Palpi yellow. Eye oval, with scattered long setae, 0.42 times as high as head and 1.9 times as high as malar space. Postgenal cushion dense. **Antennae**: A1 slightly curved, broadened apically, finely coriaceous; its apical rim simple. A2 not compressed. Apical half of A1 and A2–A8 dark brown, A9–A13 dark brown. Antenna A10–A13 with MGS brush, flattened on ventral side. A10–A12 as long as wide. A13 distinctly narrower than A12 and 1.1 times as long as A12. Antennomers length to width ratios in dorsal view as in Fig. 12A and D; A13 with small shallow ventral tip. **Mesosoma**: black, as wide as high. Neck bare, with longitudinal grooves. Pronotum with median area and pronotal corner pubescent, pronotal cushion dense; pronotal corner weakly prominent, rounded; lateral area of pronotum smooth and bare. Tegula dark brown, large. Mesopleuron smooth, shiny and bare, with subalar ridge. Sternaulus absent. Epicnemial pit tiny and bare inside. Ventral side of mesopleuron pubescent. Mesoscutum 1.25 times as wide as long, without notauli. Humeral sulcus distinct and narrow. Anterior scutellar pitscircular with short and low elongate keels posteriorly (Fig. 12B). Lateral scutellar pit broad. Posterior scutellar pits distinct. Metanotum sparse pubescent, coarsely sculptured, metascutellum with three low longitudinal keels. Propodeum pubescent and coarsely rugose, its posterior margin without arcuate emargination in dorsal view between plicae. Median propodeal keel projecting into high spine anteriorly. All legs slender, pale brown, with separated trochantelli. **Wings**: Marginal vein elongate, twice as long as its median width. Stigmal vein as wide as width of marginal vein. Costa and basal veins sclerotised, weakly pigmented. **Metasoma**: Petiole cylindrical, 1.3 times as long as wide, striate, weakly setose dorsally (with hirsute belt medially) and densely pubescent ventrally. T2 about 3.9–4.5 times as long as petiole, smooth and bare. T3–T6 and S3–S6 with few erect long setae, almost smooth (with small area of micropunctures medially). T5 weakly expanded laterally. T7 subtriangle, with long setae around spiracles. S6 pointed, more densely pubescent on the top.

**Figure 13.**
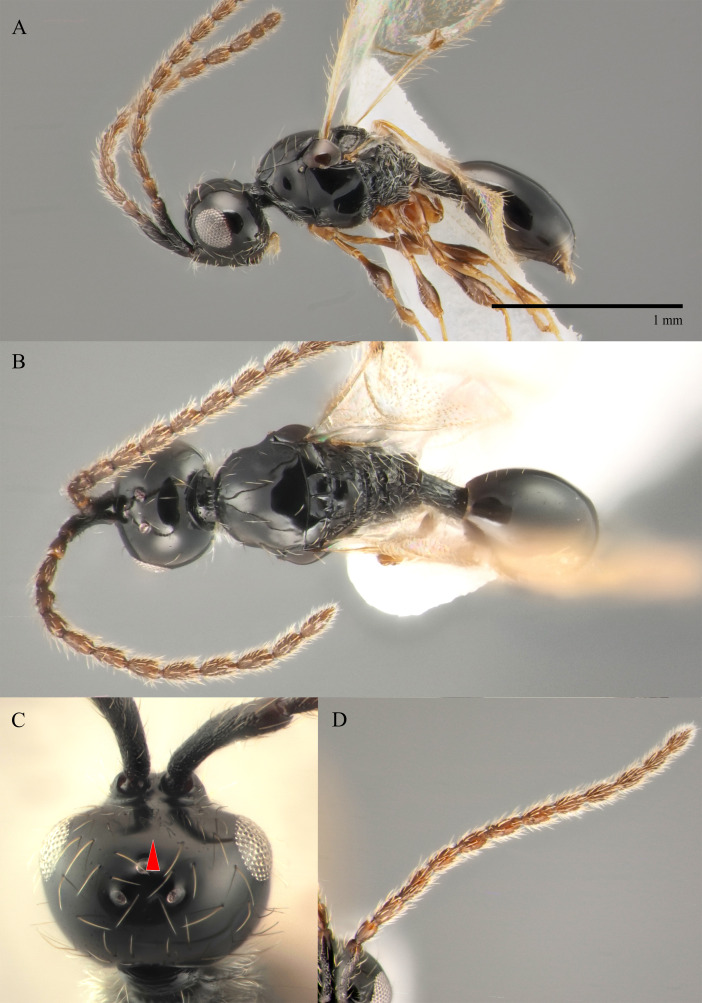
Male *Spilomicruspolitus* sp. nov. (ZSM-HYM-42318-B01; BOLD:AER1505). **A** lateral; **B** dorsal; **C** head dorsal without holes, bare area marked with red arrow; **D** antenna.

**Male (BOLD: AER1505)**. Body length 1.6 mm. Similar to female, but differs by the following features: antenna filiform, A2–A13 brown, A1 dark brown (Fig. 13A, B and D); A4 with keel running from base to 0.7 of the segment; A4 as long as A3 and 1.2 times as long as A5; A5-A10 about twice as long as wide in dorsal view; malar space 0.47 times as long as pleurostomal distance and 0.54 times as long as largest diameter of eye; petiole twice as long as wide; T2 2.8 times as long as petiole. S8 densely micropunctate.

#### Diagnosis

The species closely resembles *S.diversus* Chemyreva, 2021 from which it can be distinguished by the combination of the following features: A11 and A12 2.7 times as wide as A5 (A11–A12 about 2.3 times as wide as A5 in *S.diversus*); the malar sulcus is totally absent (visible in the form of shallow furrow in *S.diversus*); frons above base of toruli smooth (Fig. 13C) (with two small round and shallow depressions in *S.diversus*).

#### Etymology

The name of the new species is a Latin masculine adjective “politus” (smooth).

#### Distribution

Estonia, Georgia (Republic of Abkhazia and Autonomous Republic of Adjara), Germany, Romania, Russia (European part).

#### Notes

The new species *Spilomicruspolitus* sp. nov. was assigned two BINs, BOLD:ACZ2358 and BOLD:AER1505. It was not reliably possible to separate those two BINs into two morphologically sound species. The distance between those two BINs is 1.74%, whereas the distances to *Spilomicrusdiversus* (BOLD:ADF4749) are 2.59% (BOLD:ACZ2358) and 3.12 % (BOLD:AER1505), the distance to *S.modestus* is 13.6%. The fact that both BINs of the *S.politus* sp. nov. differ in under 2% of the bases in their sequences leads to the suspicion that the specimens might just be one species.

### Spilomicrus rufitarsis

Kieffer, 1911

ABA63614-62F4-50DB-891D-541E8CAA3613

AEK1604

Spilomicrus rufitarsis Kieffer, 1911 : 786.
Spilomicrus pseudocursor Szabo, 1974 : 497. Synonymised by Chemyreva (2021).

#### Description

Illustrated in Chemyreva (2021): fig. 15.

#### Distribution

Algeria, Austria, Czech Republic, France, Germany, Hungary, Ireland, Italy, Netherlands, United Kingdom.

### Spilomicrus stigmaticalis

Westwood, 1832

8B0D8C7F-AF19-58B9-B3A8-D9F30785CC28

ADS1706

ACU1243

**Figure 14.**
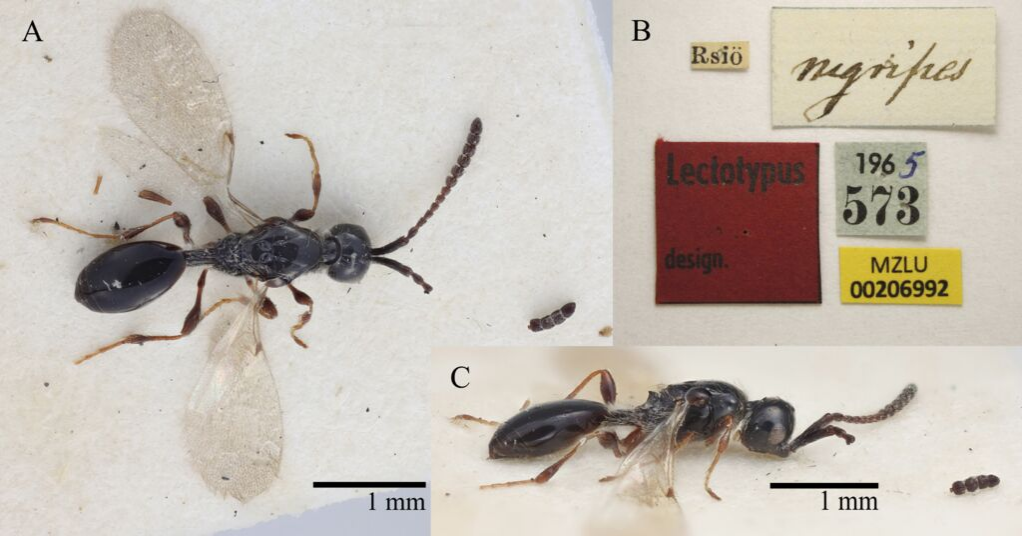
Female lectotype of the *Spilomicrusnigripes* Thomson, 1858. **A** dorsal; **B** corresponding labels; **C** lateral.

Spilomicrus stigmaticalis Westwood, 1832 : 129, female.
Spilomicrus nigripes Thomson, 1859. Synonymised by Nixon (1980). Fig. 14.
Spilomicrus basalyformis Marshall, 1868. Synonymised by Chemyreva (2021).
Spilomicrus armatus Ashmead, 1893. Synonymised by Masner (1991).
Spilomicrus tripartitus Kieffer, 1911. Synonymised by Nixon (1980).
Spilomicrus pilicornis Szabo, 1977b. Synonymised by Chemyreva (2021).
Spilomicrus barbatus Szabo, 1983. Synonymised by Chemyreva (2021).
Spilomicrus mediofurcatus Szabo, 1983. Synonymised by Chemyreva (2021).

#### Materials

- **Type status:** Lectotype. **Occurrence:** catalogNumber: MZLU 00206992; recordedBy: Thomson; individualCount: 1; sex: female; lifeStage: adult; otherCatalogNumbers: BOLD:ACU1243; occurrenceID: 1BA744EC-3DAD-5F67-BA17-27BF144BA292; **Taxon:** scientificName: Spilomicrusnigripes, Thomson, 1859; kingdom: Animalia; phylum: Arthropoda; class: Insecta; order: Hymenoptera; family: Diapriidae; genus: Spilomicrus; specificEpithet: stigmaticalis; scientificNameAuthorship: Westwood, 1832; **Location:** continent: Europe; country: Sweden; locality: Ringsjon in Skĺne; **Identification:** identifiedBy: V. Chemyreva; dateIdentified: 2023; identificationRemarks: designated here, Fig. 14; **Event:** eventDate: 1965; **Record Level:** ownerInstitutionCode: MZLU

#### Distribution

Algeria, Azerbaijan, Austria, Canada, Czech Republic, Finland, France, Georgia, Germany, Hungary, Ireland, Italy, Kazakhstan, Moldova, Netherlands, Poland, Russia (European part and Siberia), Slovakia, Sweden, Switzerland, Ukranie, United Kingdom, United States.

#### Notes

*Spilomicrusstigmaticalis* is a fairly common, widely distributed species. The species contains two BINs, BOLD:ADS1706 and BOLD:ACU1243. Still, all sequences are clustered as one single taxon using the BOLD cluster analysis and the ASAP algorithm. Not only is the genetic distance between those BINs small (1.9%), they also show medium to high intraspecific variation of up to 2.2% (mean distance 0.6%). In addition to that, we were not able to distinguish both genetic clades morphologically in both sexes, not even using the genitalia. It was only possible to find identifying morphological characters to distinguish between the females. Due to the genetic and morphological proximity of both clades, we will keep them together as one species. A lectotype is designated for *Spilomicrusnigripes* Thomson, 1858 (Fig. 14).

### Spilomicrus thomsoni

Kieffer, 1911

ED2020B5-F052-58D8-8A03-900C011E121A

ADF4747

ADX1651

Spilomicrus thomsoni Kieffer, 1911 : 787, 798.

#### Materials

- **Type status:** Lectotype. **Occurrence:** recordedBy: C. H. Boheman; individualCount: 1; sex: female; lifeStage: adult; occurrenceID: 051550F1-0B2D-5654-9E40-7E0ED1AF0AA2; **Taxon:** scientificName: Spilomicrusthomsoni; kingdom: Animalia; phylum: Arthropoda; class: Insecta; order: Hymenoptera; family: Diapriidae; genus: Spilomicrus; specificEpithet: thomsoni; scientificNameAuthorship: Kieffer, 1911; **Location:** continent: Europe; country: Sweden; stateProvince: Smĺland; **Record Level:** institutionCode: NHRS-HEVA; collectionCode: 000016369; ownerInstitutionCode: NHRS; source: designated by Chemyreva 2021

#### Diagnosis

**Figure 15.**
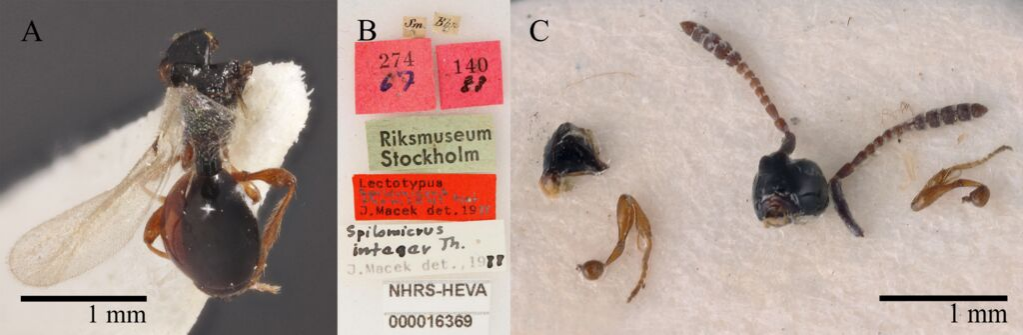
Female lectotype of *Spilomicrusthomsoni*. **A** dorsal; **B** corresponding labels; **C** broken off body parts.

Malar sulcus partly developed, shallow; neck of prothorax bare anteriorly; pleurostomal distance distinctly shorter than distance between eyes in front view; temples behind eyes gradually receding posteriorly; male А4 cylindrical, with keel reaching 0.55 of the segment length, A4 0.73–0.80 times as long as A3; antenna gradually darkened towards the top, with non-abrupt clava; female A9 with distinct MGS brush; A13 with small pit ventrally; A13 in dorsal and lateral views narrower than A12; А9 distinctly narrower and shorter than А10; notauli present in the form of broad grooves posteriorly; sternaulus absent; wings reaching to apex of metasoma to distinctly beyond it; female petiole elongate, about 1.2 times as long as wide. Lectotype illustrated in Fig. 15.

#### Distribution

Czech Republic (Tomsik 1947), Finland, Germany*, Moldova, Russia (European part), Sweden, Ukraine.

#### Notes

There are two BINs within *Spilomicrusthomsoni*, BOLD:ADF4747 and BOLD:ADX1651, which differ in only 0.1% from each other. Although the cluster methods of ASAP and BOLD separate the two clades and show very low intraspecific genetic variation, we could not tell them morphologically apart. Therefore, we will refer to them as being one species until further analyses might change that interpretation.

On the other hand, we can separate the *Spilomicrusthomsoni* taxon from *S.hemipterus* genetically and morphologically. This is why we removed *S.thomsoni* from synonymy with *S.hemipterus*.

## Identification Keys

### Key to the European *Spilomicrus* species (modified and updated after Chemyreva (2021))

**Table:** 

1	Ventral margin of clypeus with pointed or rounded deflexed median projection; mandibles short, with upper tooth much shorter than lower tooth	2
–	Ventral margin of clypeus with rounded reflexed median projection (fig. 17, *2* [arrow], *4* in Chemyreva (2021)); mandibles elongate, with upper tooth only slightly shorter than lower tooth (fig. 17, *4* in Chemyreva (2021))	4
2	Antennae with clava 5- or 6-segmented; in front view, ventral margin of clypeus rounded, blunt; mesosoma depressed, no more than 0.8 times as high as wide, mesoscutum weakly convex; median propodeal keel low, hardly raised anteriorly	***S.sanbornei***
–	Antennae with clava 7- or 8-segmented; in front view, ventral margin of clypeus triangular, pointed; mesosoma less depressed, at least 0.9 times as high as wide, mesoscutum strongly convex; median propodeal keel distinctly raised anteriorly to form a high projection	3
3	Antennal clava 7-segmented, A8–A12 strongly transverse; notauli weakly convergent anteriorly or subparallel, developed in posterior fourth or absent	***S.crassiclavis***
–	Antennal clava 8-segmented, A8–A12 subquadrate or elongate; notauli distinctly divergent anteriorly and always developed at least in posterior third	***S.formosus***
4	(1) All femora broad, with very short stalks (fig. 5, *9*; fig. 11, *9*; fig. 14, *2* in Chemyreva (2021)); clypeus more than twice as wide as high (fig. 5, *2*; fig. 11, *1*; fig. 14, *5*; fig. 15, *2* in Chemyreva (2021))	5
–	All femora slender, with long stalks (fig. 8, *1*, *8* in Chemyreva (2021)); clypeus less than twice as wide as high (fig. 1, *2*; fig. 2, *1*; fig. 4, *3* in Chemyreva (2021)) [except *S.stigmaticalis*]	8
5	Antenna with abrupt 6-segmented clava, A3–A7 yellowish, A8–A13 dark brown (fig. 5, *4*, *5* in Chemyreva (2021)); hind femur longitudinally deeply grooved (with distinct sharp margins) on ventral side for reception of tibia (fig. 5, *7* in Chemyreva (2021))	***S.compressus***
–	Antenna with non-abrupt clava, uniformly reddish-brown to black (fig. 11, *5*; fig. 14, *4* and fig. 15, *3* in Chemyreva (2021)); hind femur with smooth bare area or shallow depression on ventral side or not modified	6
6	Clava slender, A11 about 1.5 times as wide as A4 in dorsal view and about 1.25 times, in lateral view (fig. 14, *4* in Chemyreva (2021)); notauli developed in posterior half and narrow throughout (fig. 14, *3* in Chemyreva (2021))	***S.nigriclavis***
–	Clava wider, A11 about twice as wide as A4 in dorsal view and about 1.75 times, in lateral view (fig. 11, *5* and fig. 15, *3*, *4* in Chemyreva (2021)); notauli developed only in the form of oval or round posterior point or (if they are longer) distinctly broadened posteriorly (fig. 11, *4* and fig. 15, *6* in Chemyreva (2021)), sometimes completely absent	7
7	Neck of prothorax with short longitudinal grooves posteriorly; notauli developed at least in posterior third of mesoscutum (fig. 15, *6* in Chemyreva (2021)); propodeum with median keel strongly raised anteriorly (fig. 15, *6* in Chemyreva (2021)); A13 as long as A12	***S.rufitarsis***
–	Neck of prothorax entirely smooth (fig. 11, *3* in Chemyreva (2021)); notauli developed on mesoscutum only in the form of small posterior pits to completely absent (fig. 11, *4* in Chemyreva (2021)); propodeum with median keel slightly raised anteriorly (fig. 11, *4* in Chemyreva (2021)); A13 about 1.3–1.4 times as longer A12	***S.latus***
8	(4). Base of T2 pubescent (fig. 3, *3* and fig. 12, *4* in Chemyreva (2021))	9
–	Base of T2 bare (fig. 1, *4* in Chemyreva (2021))	11
9	Micropterous (fig. 1 C. fig. 3 in Chemyreva (2021)); T2 with scattered long setae (fig. 3, *1*, *3* in Chemyreva (2021)); scutellum strongly transverse, without posterior scutellar pits (fig. 3, *3* in Chemyreva (2021)); head subquadrate in dorsal view (fig. 3, *2* in Chemyreva (2021)); ocelli absent	***S.antennatus***
–	Macropterous (Fig. 3A); T2 bare (fig. 12, *2* in Chemyreva (2021)); scutellum slightly transverse to elongate, with posterior scutellar pits (fig. 12, *4* in Chemyreva (2021)); head transverse in dorsal view; ocelli present	10
10	Propodeum with deep emargination between plicae, plicae slightly convergent posteriorly (Fig. 3B)	***S.brevimalaris* sp. nov.**
–	Propodeum with not deep emargination between plicae, plicae not convergent posteriorly (Fig. 8B)	***S.flavecorpus* sp. nov.**
11	(8). T2 with numerous scattered long setae (fig. 6, *1* in Chemyreva (2021)); two posterior ocelli absent (fig. 6, *4* in Chemyreva (2021))	***S.cursor***
–	T2 bare; all ocelli present	12
12	Propodeum with deep arcuate emargination of posterior margin between plicae in dorsal view (fig. 2, *2*; fig. 9, *6*; fig. 10, *8* and fig. 17, *3* in Chemyreva (2021)); body mainly larger than 2.0 mm	13
–	Propodeum with weak arcuate emargination of posterior margin between plicae in dorsal view (fig. 1, *4*; fig. 7, *1* and fig. 13, *4* in Chemyreva (2021)); body mainly smaller than 2.0 mm	19
13	Sternaulus complete (fig. 17, *1* in Chemyreva (2021)); A13 without pit ventrally; A13 in dorsal and lateral views not narrower than A12; clava elongate, А9 as wide and as long as А10 [not always in *S.flavipes*] (fig. 2, *3* and fig. 17, *5*, *6* in Chemyreva 2021)	14
–	Sternaulus absent at least medially (fig. 9, *7* in Chemyreva (2021)); A13 with distinct small pit ventrally; A13 in dorsal and lateral views narrower than A12; clava fusiform [not always in *S.hemipterus*], А9 distinctly narrower and shorter than А10 (fig. 4, *5*, *6*; fig. 9, *3*, *4* and fig. 10, *3*, *5* in Chemyreva (2021))	17
14	Head in front view with transverse wrinkles on antennal shelf (fig. 8, *2* in Chemyreva (2021)); temples distinctly, but gradually receding behind eyes in dorsal view (fig. 8, *5* in Chemyreva (2021))	***S.flavipes***
–	Head in front view without wrinkles on antennal shelf (fig. 2, *1* and fig. 17, *4* in Chemyreva (2021)); temples parallel behind eyes in dorsal view (fig. 2, *4* and fig. 17, *7* in Chemyreva (2021))	15
15	А3–А6 pale brown and clava black; tentorial pit absent to very tiny (punctiform) (fig. 2, *1* in Chemyreva (2021)); scutellum parallel-sided to narrowed posteriorly (fig. 2, *2* in Chemyreva 2021); А3 1.5 times as long as А2 (fig. 2, *3* in Chemyreva (2021))	***S.annulicornis***
–	А1–А13 black; tentorial pit distinct (Fig. 16C); scutellum slightly broadened posteriorly (Fig. 16D); А3 equal to 1.2 times as long as A2 (Fig. 16E)	***S.stigmaticalis***
16	(13). Notauli usually absent, when rarely present, then expressed only in the form of two narrow incisions; malar sulcus totally absent	***S.integer***
–	Notauli present in the form of broad grooves posteriorly; malar sulcus present, partly developed or fully visible in the form of a shallow groove	17
17	Neck of prothorax pubescent anteriorly (fig. 4, *7* in Chemyreva (2021)); pleurostomal distance distinctly longer than distance between eyes in front view (fig. 4, *3* in Chemyreva (2021)); temples behind eyes parallel or even weakly divergent posteriorly in dorsal view (fig. 4, *7* in Chemyreva (2021))	***S.bipunctatus***
–	Neck of prothorax bare anteriorly (fig. 9, *6* in Chemyreva (2021)); pleurostomal distance distinctly shorter than distance between eyes in front view (Fig. 17, Fig. 18C); temples behind eyes gradually receding posteriorly	18
18	Female antennae distinctly bicolor with abrupt 5-segmented clava (Fig. 17D); A10–A13 with MGS brush (all multiporous gustatory sensillae on the antenna) on its ventral side	***S.hemipterus***
–	Female antennae more or less monochrome with non-abrupt clava (Fig. 18D); A9–A13 with MGS brush on its ventral side	***S.thomsoni***
19	(12). Notauli in the form of short grooves on mesoscutum posteriorly (fig. 1, *4* in Chemyreva (2021)); malar sulcus not deep, but completely developed throughout (fig. 1, *2* in Chemyreva (2021))	***S.abnormis***
–	Notauli totally absent; malar sulcus absent or incompletely developed (fig. 7, *2*, *5* and fig. 13, *2* in Chemyreva (2021))	20
20	Head in dorsal view with temples parallel behind eyes (fig. 13, *5* in Chemyreva (2021)); petiole subquadrate to transverse (fig. 13, *4* in Chemyreva (2021)); antennae entirely brown, moniliform, without clava (fig. 13, *3* in Chemyreva (2021))	***S.modestus***
–	Head in dorsal view with temples receding behind eyes (fig. 7, *8* in Chemyreva (2021)); petiole slightly elongate to 1.8 times as long as wide (Fig. 12B, Fig. 6B); antennae with dark abrupt 5-segmented clava, A2–A8 pale brown (Figs 6, 12D)	21
21	Front behind scapus with two small oval and not deep holes (as in Fig. 5C)	***S.diversus***
–	Front behind scapus smooth (as in Fig. 13C)	***S.politus* sp. nov.**

**Females**

(female of *S.lusitanicus* unknown)

### Males (males of *S.cursor* and *S.nigriclavis* unknown)

**Table:** 

1	Ventral margin of clypeus with pointed or rounded deflexed median projection; mandibles short, with upper tooth much shorter than lower tooth	2
–	Ventral margin of clypeus with small rounded reflexed median projection (fig. 17, *2* [arrow], *4* in Chemyreva (2021)); mandibles elongate, with upper tooth slightly shorter than lower tooth	4
2	In front view, ventral margin of clypeus rounded, blunt; A4 with moderately deep, curved emargination; mesosoma distinctly depressed, no more than 0.8 times as high as wide, mesoscutum weakly convex; median propodeal keel low, hardly raised anteriorly	***S.sanbornei***
–	In front view, ventral margin of clypeus triangular, acuminate; A4 with at most a shallow emargination; mesosoma less depressed, at least 0.9 times as high as wide, mesoscutum strongly convex; median propodeal keel raised anteriorly to form a high projection	3
3	Eye sparsely hairy; А4 with carina over-reaching 0.7 of the segment	***S.crassiclavis***
–	Eye bare; А4 with carina not over-reaching basal half of the segment	***S.formosus***
4	(1). Clypeus transverse, more than twice as wide as high (fig. 5, *2*; fig. 11, *1* and fig. 15, *2* in Chemyreva (2021))	5
–	Clypeus rounded, less than twice as wide as high (fig. 1, *2*; fig. 2, *1* and fig. 4, *3* in Chemyreva (2021))	8
5	A4 distinctly longer than A3	***S.stigmaticalis***
–	A4 distinctly shorter than A3	6
6	A5–A12 at least twice as long as wide (fig. 5, *3* in Chemyreva (2021)); legs yellowish-brown	***S.compressus***
–	A5–A12 at most 1.5 times as long as wide (fig. 11, *6* and fig. 15, *5* in Chemyreva (2021)); legs dark brown	7
7	Neck with short longitudinal grooves posteriorly; notauli developed at least in posterior half of mesoscutum (fig. 15, *6* in Chemyreva (2021)); propodeum with median keel strongly raised anteriorly; A3 1.1–1.3 times as long as A4 (fig. 15, *5* in Chemyreva (2021))	***S.rufitarsis***
–	Neck entirely smooth (fig. 11, *3* in Chemyreva (2021)); notauli developed on mesoscutum in the form of small pits posteriorly to absent (fig. 11, *4* in Chemyreva (2021)); propodeum with median keel slightly raised anteriorly; A3 1.5–1.6 times as long as A4 (fig. 11, *8*Chemyreva (2021))	***S.latus***
8	(4). Base of T2 pubescent (fig. 3, *3*; fig. 12, *4* in Chemyreva (2021))	9
–	Base of T2 bare (fig. 1, *4* in Chemyreva (2021))	12
9	A4 without emargination and keel (fig. 16, *6* in Chemyreva (2021))	***S.antennatus***
–	A4 with emargination and keel (fig. 12, *8*, *9* in Chemyreva (2021))	10
10	Malar space 0.2–0.22 times as long as largest diameter of eye (Fig. 4B) and 0.24–0.27 times as long as distance between pleurostoma	***S.brevimalaris* sp. nov.**
–	Malar space more than 0.42 times as long as largest diameter of eye (Fig. 4A and C) and 0.40–0.45 times as long as distance between pleurostoma	11
11	Head narrower than mesosoma (Fig. 11B); A5–A12 more than 2.3 times as long as wide (Fig. 11C and D); scutellum as long as wide or distinctly elongated	***S.lusitanicus***
–	Head as wide as to wider than mesosoma in dorsal view (Fig. 7B); A5–A12 about 1.3 times as long as wide (Fig. 7A and C); scutellum distinctly transverse	***S.flavecorpus* sp. nov.**
12	(8). A3 distinctly longer than А4 (fig. 4, *2*; fig. 9, *8* and fig. 10, *6*, *7* in Chemyreva (2021))	13
–	А3 as long as or shorter than А4 (fig. 1, *7*; fig. 2, *7*; fig. 7, *4*; fig. 12, *9* and fig. 17, *10* in Chemyreva (2021))	16
13	Notauli present (fig. 4, *1* and fig. 9, *6* in Chemyreva (2021)); keel on A4 not reaching apex of the segment (fig. 4, *2* and fig. 9, *8* in Chemyreva (2021)); malar sulcus present (partly developed or fully visible in the form of a shallow groove)	14
–	Notauli absent (fig. 10, *8* in Chemyreva (2021)); keel on А4 reaching apex of the segment (fig. 10, *6* in Chemyreva (2021)); malar sulcus totally absent (fig. 10, *2* in Chemyreva (2021))	***S.integer***
14	Neck of prothorax pubescent anteriorly (fig. 4, *7* in Chemyreva (2021)); pleurostomal distance distinctly longer than distance between eyes in front view (fig. 4, *3* in Chemyreva (2021)); temples behind eyes parallel or even divergent posteriorly in dorsal view (fig. 4, *7* in Chemyreva (2021))	***S.bipunctatus***
–	Neck of prothorax bare anteriorly (fig. 9, *5*, *6* in Chemyreva (2021)); pleurostomal distance distinctly shorter than distance between eyes in front view (fig. 9, *2* in Chemyreva (2021)); temples behind eyes usually convergent posteriorly (fig. 9, *5* in Chemyreva (2021))	15
15	A4 0.65 times as long as A3, widened apically with keel reaching 0.7 of the segment length (Fig. 19a)	***S.hemipterus***
–	A4 0.73 times as long as A3, cylindrical with keel reaching 0.55 of the segment length (Fig. 19b)	***S.thomsoni***
16	(12). А4 with projection at base of keel and with bare smooth area along this keel (fig. 2, *7*; fig. 8, *6*, *7* and fig.17, *10* in Chemyreva (2021)); sternaulus complete; body usually longer than 2.0 mm	17
–	А4 without projection at base of keel and without bare smooth area along this keel (fig. 1, *7*; fig. 7, *4* and fig. 13, *6* in Chemyreva (2021)); sternaulus absent medially; body usually shorter than 2.0 mm	19
17	Propodeum with deep arcuate emargination of posterior margin between plicae in dorsal view (fig. 8, *8* and fig. 17, *3* in Chemyreva (2021)); А3–А5 in lateral view equal to each other in width; pubescence of A3–A13 less dense, semi-erect (fig. 8, *6* and fig. 17, *9* in Chemyreva (2021))	12
–	Propodeum with weak arcuate emargination of posterior margin between plicae in dorsal view; А4 in lateral view wider than А3 and А5; pubescence of А3–А13 more dense, recumbent (fig. 2, *7* in Chemyreva (2021))	***S.annulicornis***
18	Head in front view with transverse wrinkles on the top of antennal shelf (fig. 8, *2*); antenna pale brown to brown, emargination on А4 shallow (fig. 8, *6* in Chemyreva (2021)); mesoscutum smooth anteriorly and with notauli developed in posterior half	***S.flavipes***
–	Head in front view without transverse wrinkles on the top of antennal shelf (fig. 17, *4* in Chemyreva (2021)); antennae dark brown to black, emargination on А4 deep (Fig. 20A, B. fig. 17, *10* in Chemyreva (2021)); mesoscutum with notauli completely developed throughout, shallow anteriorly (Fig. 20B. fig. 17, *7* in Chemyreva (2021))	***S.stigmaticalis***
19	(16). Notauli developed in the form of short posterior grooves (fig. 1, *4* in Chemyreva (2021)); malar sulcus complete, shallow (fig. 1, *2* in Chemyreva (2021))	***S.abnormis***
–	Notauli totally absent (fig. 7, *1* and fig. 13, *4* in Chemyreva (2021)); malar sulcus absent (fig. 7, *2*, *5* and fig. 13, *2* in Chemyreva (2021))	20
20	Head in dorsal view subrectangular, with temples parallel behind eyes (fig. 13, *5* in Chemyreva (2021)); A5–A12 1.1–1.3 times as long as wide (fig. 13, *6* in Chemyreva (2021)); petiole subquadrate to weakly elongate	***S.modestus***
–	Head in dorsal view with temples receding behind eyes (fig. 7, *8* in Chemyreva (2021)); A5–A12 about twice as long as wide (fig. 7, *4* in Chemyreva (2021)); petiole elongate, at least 1.5 times as long as wide	21
21	Front behind scapus with two small oval and not deep holes (Fig. 5C)	***S.diversus***
–	Front behind scapus smooth (Fig. 13C)	***S.politus* sp. nov.**

## Discussion

DNA barcoding is revolutionising taxonomy research, especially when researchers are dealing with hyper- and cryptic-diverse insect taxa of small body size and variable morphological characters (Fernandez-Triana 2022). Although DNA barcoding is a great tool at hand, it has its own limitations. Various researchers (see Meier et al. (2006), Raupach et al. (2016), Ferrer-Suay et al. (2018) and Pollmann et al. (2023)) have attempted to examine the accuracy of DNA barcodes for species identification and have found discrepancies in the results depending on the targeted taxon. Heteroplasmy, NUMTs, hybridisation, recent speciation, phylogeographic effects, introgression and/or incomplete lineage sorting, endosymbionts and their combinations can all have an effect on sorting of genetic material, as well as simply high variation in the (mostly used) mitochondrial genes. Analysing different (nucleic) loci can equalise some challenges like multiple gene copies (NUMTs) and can help to interpret the actual taxonomic reality more reliably. Still, one of the major difficulties to assign a new BIN is the threshold value of difference between two sequences (usually 2% variance in the CO1 sequence). While some species can have intraspecific variation of up to 9.6% (Huemer et al. 2014) and are still considered to be one valid species, other taxa show the opposite: for example, the geometrid taxa *Boudinotiananotha* and *B.touranginii* are known to be two clearly separated species, though both share the same Barcode (Hausmann et al. 2013). It is also worth mentioning that the BIN system is dynamic and that BINs can change over time, depending on the amount of data available. Using an integrative approach, traditional morphology in combination with genetic analyses provides the opportunity to obtain a more accurate hypothesis on the taxonomic status of a taxon. Our study found evidence that the just recently described *Spilomicrusdiversus* Chemyreva, 2021 is, indeed, composed of at least two species. Although we were able to assign a BIN to the described species, *S.diversus*, *S.politus* sp. nov., on the other hand, received two BINs which only differ in 1.74% of the sequences within our dataset. The slim molecular variation in combination with a lack of morphological characters led us to the hypothesis that the two BINs align both with the same species. Therefore, we described only one new taxon, *S.politus*, with the corresponding BINs (BOLD:AER1505 and BOLD:ACZ2358). When first described in 2021, the species *S.diversus* was known to show a highly diverse morphology, as the name suggests. As a consequence of our barcoding results, one paratype had to be excluded from the series.

Another questionable case we faced was *Spilomicrusstigmaticalis* Westwood. While only an insufficient difference could be examined between the two haplotypes of the female, the males could not be distinguished morphologically at all. Interspecific variation was detected to be relatively low, while the intraspecific variation was rather high. Incomplete lineage sorting might be a reason for that, since allopatric/geographic factors, as well as seasonality could be ruled out. Taking both factors, genetics and morphology, into account, we decided to keep those two BINs in one species, *S.stigmaticalis*.

On the other hand, *S.thomsoni* was a relatively clear case. The two BINs (BOLD:ADF4747, BOLD:ADX1651), corresponding with the morphological determination, were genetically close (0.1%), while the taxon could be separated from *S.hemipterus* both genetically and morphologically.

There are still many taxonomic questions remaining regarding the Palearctic species of *Spilomicrus*. The high level of the sexual dimorphism in *S.crassiclavis* (Notton 1999) (only males were included in the current research) and the genetic relatedness of the species reported from Europe and North America (*S.antennatus*) or from the Western Palaearctic and the Eastern Palaearctic (*S.formosus*, *S.crassiclavis*, *S.abnormis*, *S.diversus* and *S.flavipes*) have not been verified yet. It has to be noted that a tree, based on a CO1-barcode alone, cannot be expected to resolve "deep" nodes correctly. Therefore, it is not suprising that, for example, the formosus species-group does not appear monophyletic (and if it is, indeed, monophyletic Notton (1999)). Additionally, as is true for many diaprid species, there are not too many host records.

## Supplementary Material

XML Treatment for Spilomicrus

XML Treatment for Spilomicrus abnormis

XML Treatment for Spilomicrus annulicornis

XML Treatment for Spilomicrus antennatus

XML Treatment for Spilomicrus bipunctatus

XML Treatment for Spilomicrus brevimalaris

XML Treatment for Spilomicrus compressus

XML Treatment for Spilomicrus crassiclavis

XML Treatment for Spilomicrus diversus

XML Treatment for Spilomicrus flavecorpus

XML Treatment for Spilomicrus flavipes

XML Treatment for Spilomicrus formosus

XML Treatment for Spilomicrus hemipterus

XML Treatment for Spilomicrus integer

XML Treatment for Spilomicrus lusitanicus

XML Treatment for Spilomicrus modestus

XML Treatment for Spilomicrus nigriclavis

XML Treatment for Spilomicrus politus

XML Treatment for Spilomicrus rufitarsis

XML Treatment for Spilomicrus stigmaticalis

XML Treatment for Spilomicrus thomsoni

0F429B85-D0BB-5196-B884-BE8C1BA93302 10.3897/BDJ.12.e114515.suppl1 Supplementary material 1 Phylogenetic ML-tree Data type taxonomic tree based on CO1 data Brief description Phylogenetic ML-tree of 45 *Spilomicrus* sequences with the outgroup *Labolipsinnupta*. File: oo_916657.svg https://binary.pensoft.net/file/916657 Huebner J. and Chemyreva V.

453A5447-1480-559A-BBF4-2457EF1C0F30 10.3897/BDJ.12.e114515.suppl2 Supplementary material 2 Table of localities Data type occurrences Brief description This table lists all the location data for each specimen that was caught within the project. Not listed are the lectotypes stored at other museums that were investigated. All the available information is printed on the labels in the image tables. File: oo_952311.xlsx https://binary.pensoft.net/file/952311 Huebner J.

## Figures and Tables

**Figure 16.**
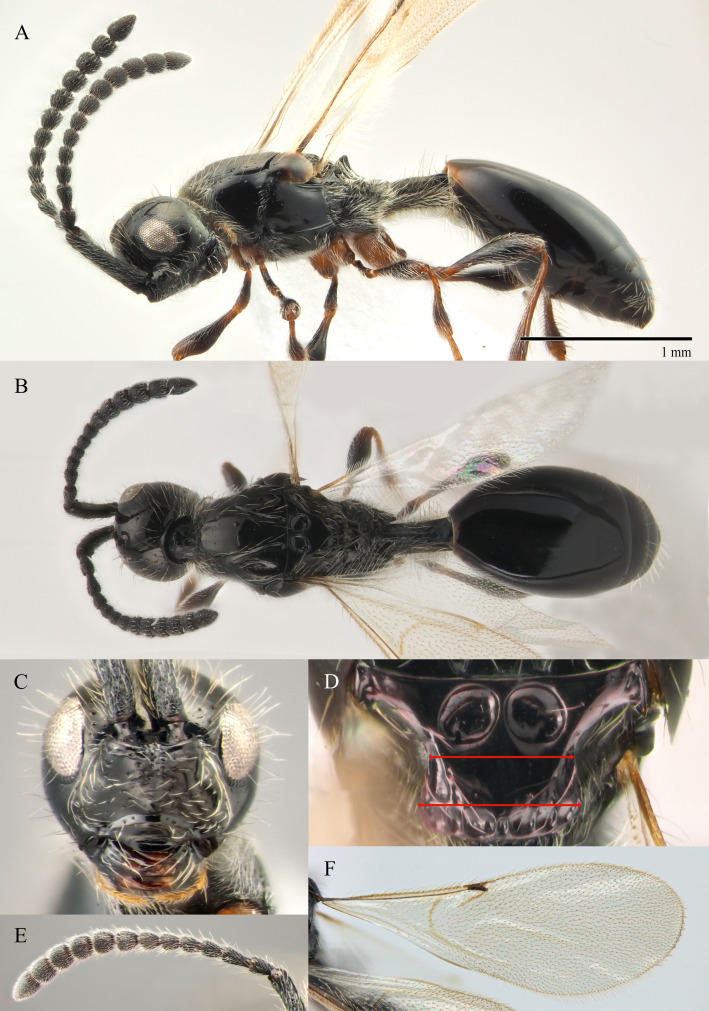
Female *Spilomicrusstigmaticalis* (ZSM-HYM-42423-H02, BOLD:ADS1706). **A** lateral; **B** dorsal; **C** face; **D** scutellum highlighted red, arrows mark the basal broadening; **E** antenna; **F** wing.

**Figure 17.**
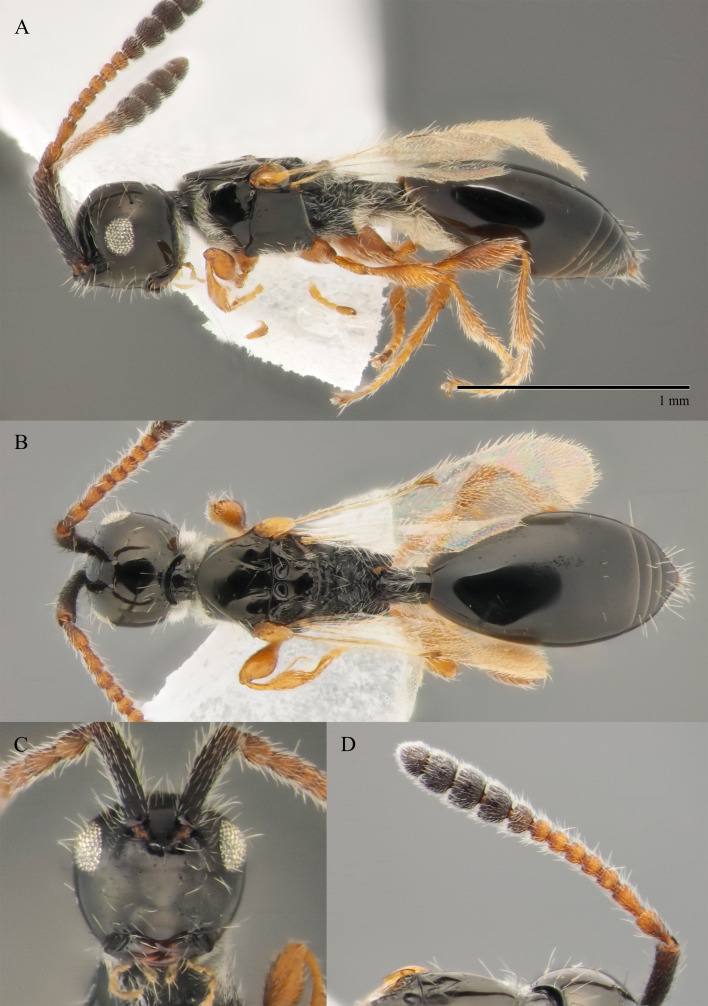
Female *Spilomicrushemipterus* (ZSM-HYM-42322-F02; BOLD:ADM6694). **A** lateral; **B** dorsal; **C** face; **D** antenna.

**Figure 18.**
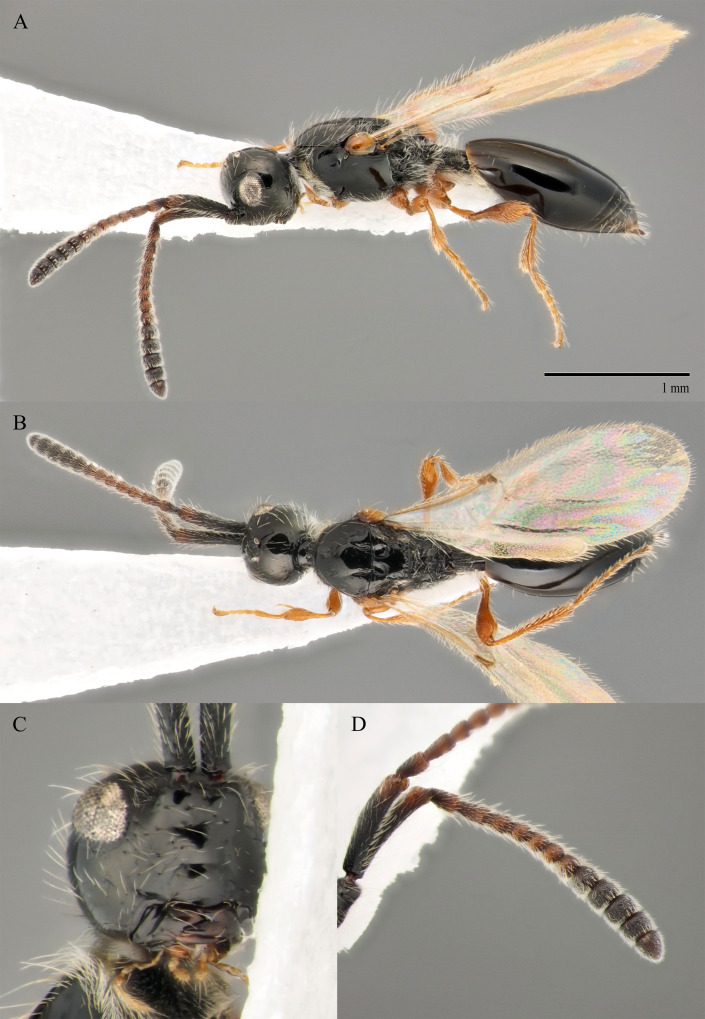
Female *Spilomicrusthomsoni* (ZSM-HYM-42321-H08; BOLD:ADF4747). **A** lateral; **B** dorsal; **C** face; **D** antenna.

**Figure 19a.**
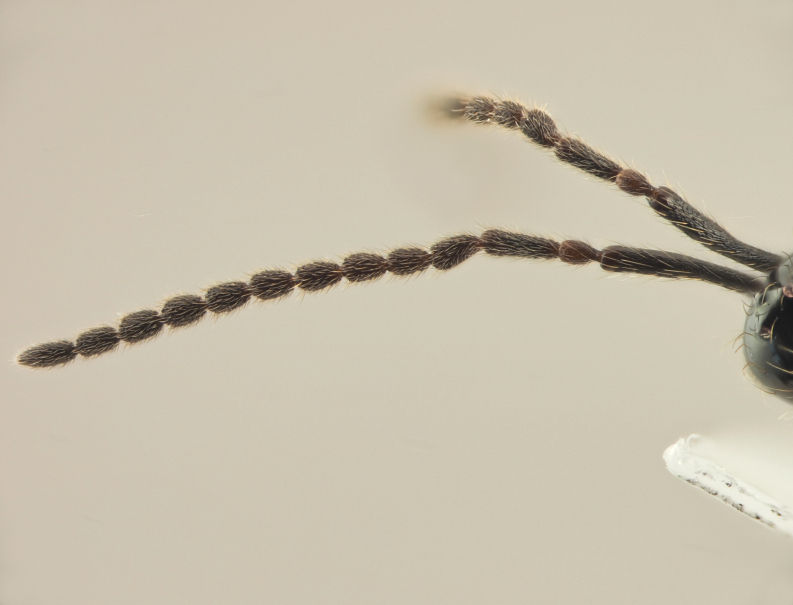
*Spilomicrushemipterus* (ZSM-HYM-42425-B06; BOLD:ADM6694);

**Figure 19b.**
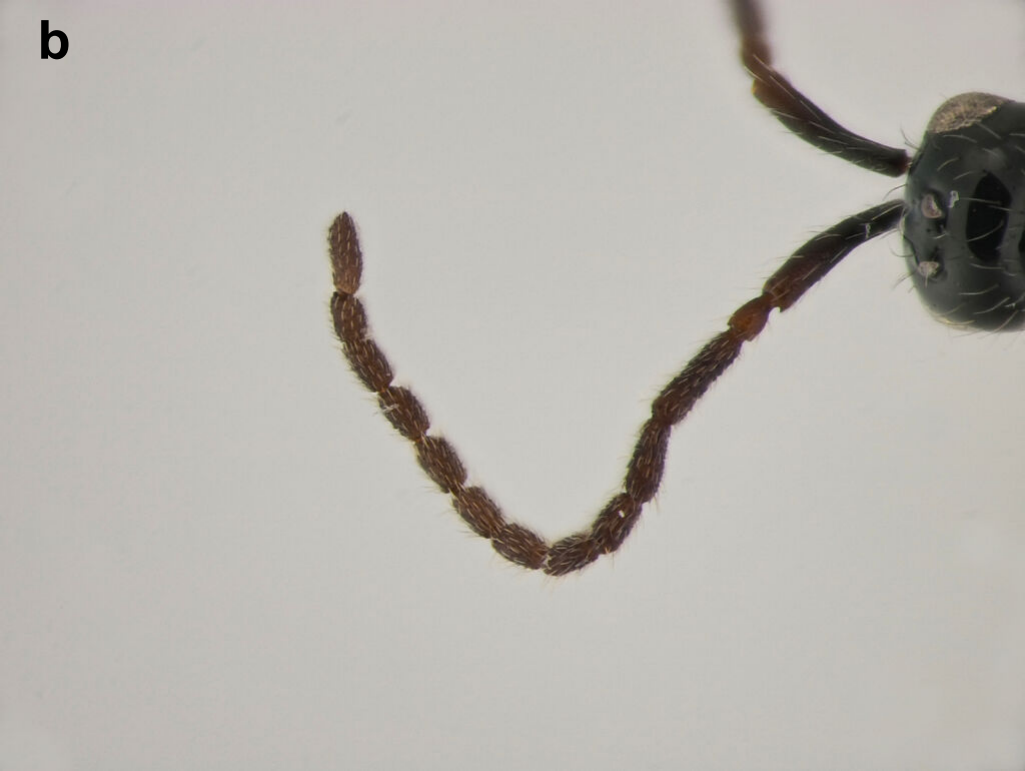
*Spilomicrusthomsoni* (ZSM-HYM-33122-A05; BOLD:ADF4747).

**Figure 20.**
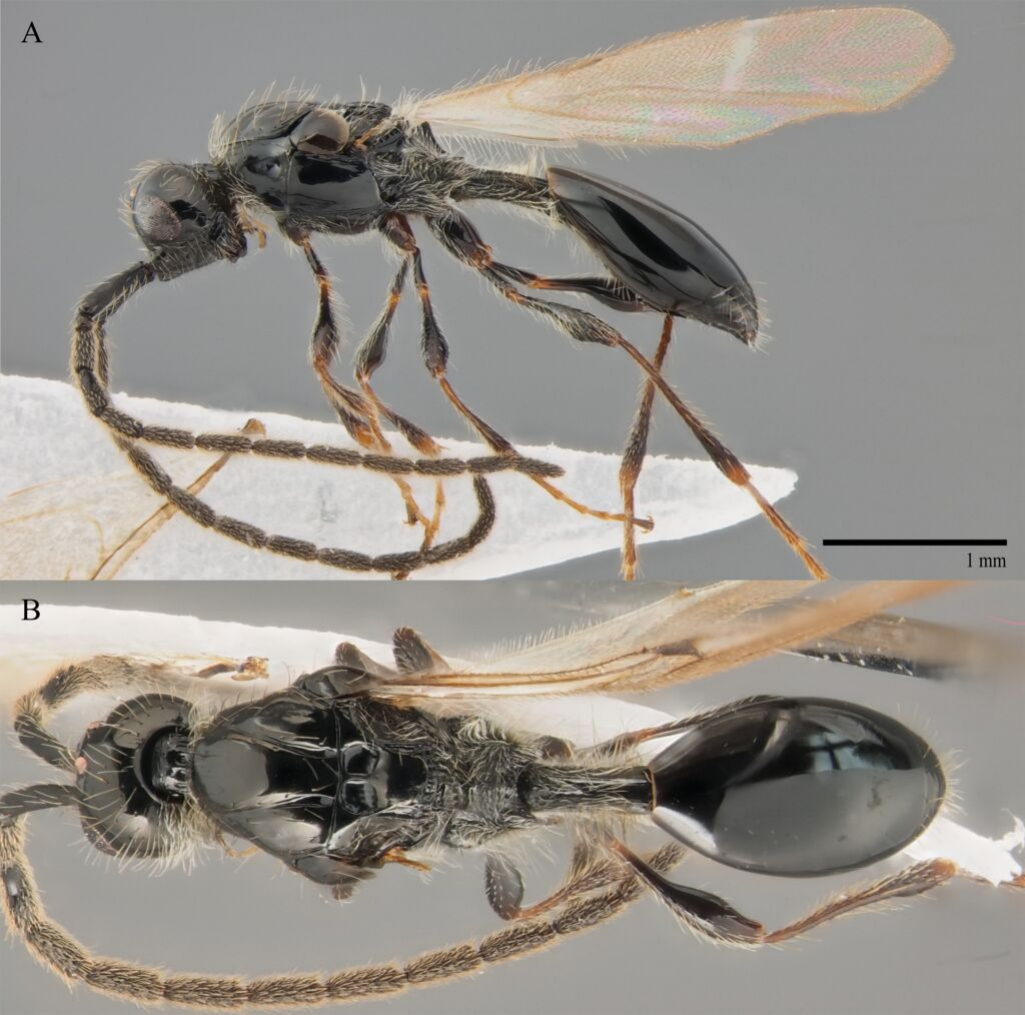
Male *Spilomicrusstigmaticalis* (ZSM-HYM-42320-F08, BOLD:ADS1706). **A** lateral; **B** dorsal.
